# Comparison of Temporal and Spatial Dynamics of Seasonal H3N2, Pandemic H1N1 and Highly Pathogenic Avian Influenza H5N1 Virus Infections in Ferrets

**DOI:** 10.1371/journal.pone.0042343

**Published:** 2012-08-08

**Authors:** Judith M. A. van den Brand, Koert J. Stittelaar, Geert van Amerongen, Leslie Reperant, Leon de Waal, Albert D. M. E. Osterhaus, Thijs Kuiken

**Affiliations:** 1 Department of Virology, Erasmus Medical Centre, Rotterdam, The Netherlands; 2 Viroclinics Biosciences B.V., Rotterdam, The Netherlands; University of Hong Kong, Hong Kong

## Abstract

Humans may be infected by different influenza A viruses—seasonal, pandemic, and zoonotic—which differ in presentation from mild upper respiratory tract disease to severe and sometimes fatal pneumonia with extra-respiratory spread. Differences in spatial and temporal dynamics of these infections are poorly understood. Therefore, we inoculated ferrets with seasonal H3N2, pandemic H1N1 (pH1N1), and highly pathogenic avian H5N1 influenza virus and performed detailed virological and pathological analyses at time points from 0.5 to 14 days post inoculation (dpi), as well as describing clinical signs and hematological parameters. H3N2 infection was restricted to the nose and peaked at 1 dpi. pH1N1 infection also peaked at 1 dpi, but occurred at similar levels throughout the respiratory tract. H5N1 infection occurred predominantly in the alveoli, where it peaked for a longer period, from 1 to 3 dpi. The associated lesions followed the same spatial distribution as virus infection, but their severity peaked between 1 and 6 days later. Neutrophil and monocyte counts in peripheral blood correlated with inflammatory cell influx in the alveoli. Of the different parameters used to measure lower respiratory tract disease, relative lung weight and affected lung tissue allowed the best quantitative distinction between the virus groups. There was extra-respiratory spread to more tissues—including the central nervous system—for H5N1 infection than for pH1N1 infection, and to none for H3N2 infection. This study shows that seasonal, pandemic, and zoonotic influenza viruses differ strongly in the spatial and temporal dynamics of infection in the respiratory tract and extra-respiratory tissues of ferrets.

## Introduction

Humans may be infected with different categories of influenza A virus—seasonal, pandemic, and zoonotic—each with their own epidemiology and pathogenesis. Seasonal influenza viruses cause annual epidemics during autumn and winter in temperate regions. They predominantly cause upper respiratory tract disease with rare extension to the lower respiratory tract, resulting in severe and even fatal pneumonia [1]. Pandemic influenza viruses, like the pandemic H1N1 (pH1N1) virus in 2009, cause sporadic pandemics with variable mortality. In fatal cases of pH1N1 infection, virus antigen expression occurred throughout the respiratory tract and was associated with both upper and lower respiratory tract disease [2]. Zoonotic influenza viruses, such as highly pathogenic avian influenza (HPAI) H5N1 virus, are sporadically transmitted from poultry and other animals to humans, but do not transmit efficiently from human to human [3]. Human infection with HPAI H5N1 virus involves primarily the lower respiratory tract, resulting in diffuse alveolar damage (DAD) and a fatality rate of almost 60% in confirmed cases [4].

The pathogenesis of both human and avian influenza virus infections in ferrets resembles that in humans [5]. In part, this is because the distribution of receptors for human and avian influenza viruses in the respiratory tract of ferrets is similar to that in humans [6]. Therefore, the ferret is often used in animal models to study the pathogenesis of different influenza virus strains and to evaluate the efficacy of vaccines and antiviral agents against influenza [7], [8], [9], [10]. In these studies, it is critical to collect respiratory tract samples for virological, pathological, and molecular analyses at both the appropriate time point after infection and the appropriate location along the respiratory tract. This is because influenza virus infection is a highly dynamic process, both temporally and spatially.

We recently compared the pathogenesis of infections with seasonal human H1N1, pH1N1, and HPAI H5N1 virus in ferrets [9]. Our results showed that, at 4 days post inoculation (dpi), pH1N1 caused pneumonia intermediate in severity between that caused by seasonal H1N1 and HPAI H5N1. This was associated with virus replication throughout the lower respiratory tract for pH1N1, while seasonal H1N1 replicated mainly in the bronchi, and HPAI H5N1 replicated mainly in the alveoli. However, the location of virus replication and extent and severity of associated pathological changes were recorded at a single time point. Other experiments have used multiple time points (usually 1, 3, 5, and 14 dpi) to study the dynamics of influenza virus infection in the ferret respiratory tract [10], [11], [12], [13], [14], [15], [16]; however, these experiments often lacked detailed pathological or virological analyses of samples collected along the full length of the respiratory tract on all time points.

The goal of our study was to describe and compare the temporal and spatial dynamics of different influenza virus infections and associated pathology in the respiratory tract of the ferret. To this end, we inoculated ferrets with either seasonal human H3N2, pH1N1, or HPAI H5N1 virus, and performed detailed virological and pathological analyses at time points from 0.5 to 14 dpi, as well as measuring virus excretion, clinical signs, and hematological parameters. Additionally, we compared the results of histopathological analyses with digital microscopical scoring of tissue sections.

## Results

### Seasonal H3N2

#### Clinical data and gross pathology

**Figure 1:**
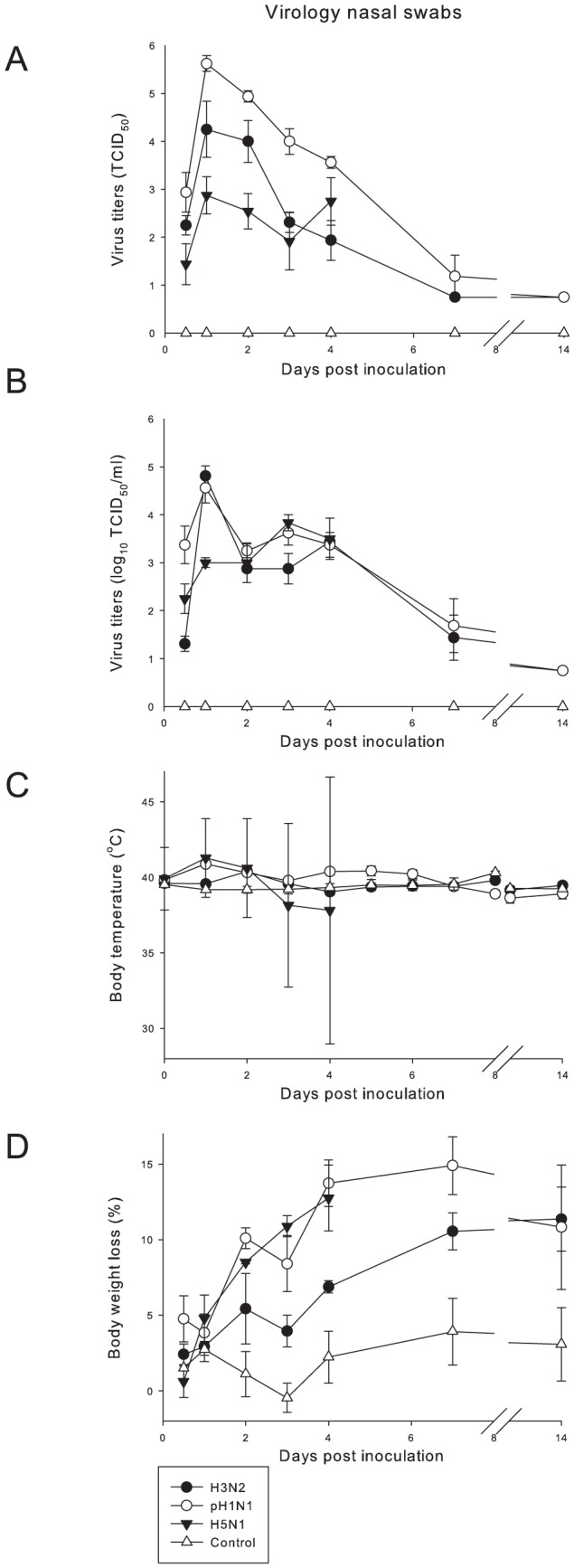
Clinical data of ferrets inoculated with different influenza viruses. The data are depicted as median values and standard errors of the mean (SEM). (A) Virus titers in nasal swabs demonstrate highest titers for pH1N1, intermediate titers for H3N2 and lowest titers for H5N1. (B) Virus titers in pharyngeal swabs demonstrate initially comparable virus titers for pH1N1 and H3N2 and lower titers for H5N1, and on 4 dpi comparable titers for pH1N1 and H5N1 and lower titers for H3N2. (C) Body temperatures in ferrets inoculated with pH1N1 and H5N1 are higher than for H3N2 and the sham-inoculated group. The large SEM for H5N1 is due to the low body temperatures of moribund animals. (D) Body weight loss in animals inoculated with pH1N1 and H5N1 was comparable and higher when compared to H3N2.

**Figure 2:**
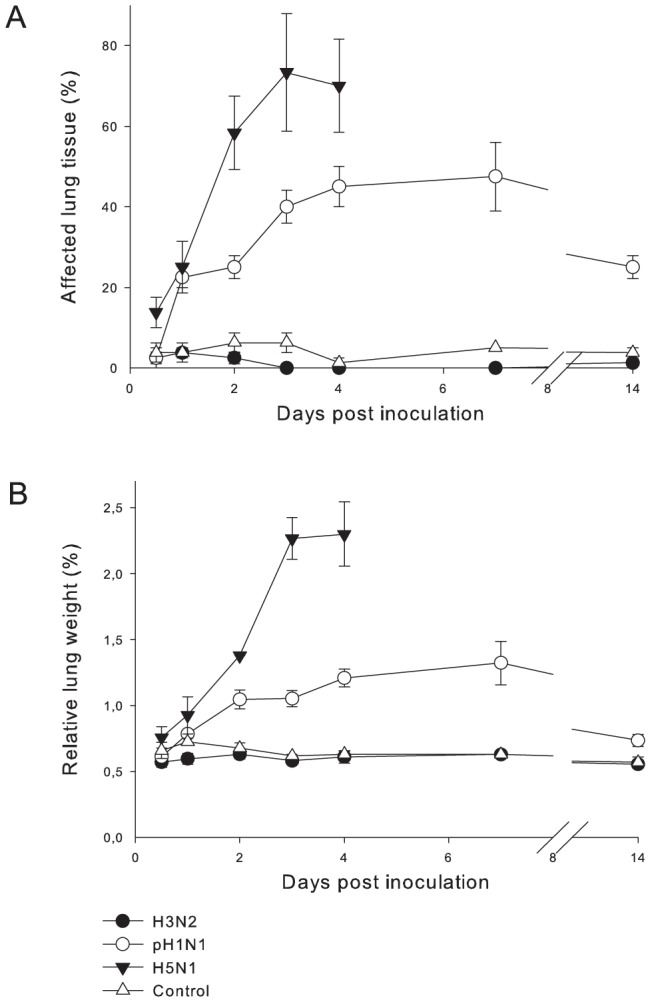
Gross pathology of the lungs of ferrets inoculated with different influenza viruses. The data are depicted as median values and standard errors of the mean. (A) Percentages of affected lung tissue show highest values for H5N1, intermediate values for pH1N1 and lowest values for H3N2. (B) Percentages relative lung weight show highest values for H5N1, intermediate values for pH1N1 and lowest values for H3N2.

The survival rate was 100% in all groups. Clinical signs (Table 1) were mild with sneezing from 2 to 14 dpi and nasal discharge from 2 to 3 dpi, as shown by increased licking on the nose. Nasal and pharyngeal swabs revealed excretion of virus from 0.5 to 4 dpi with a peak on 1 dpi in the nasal swabs (Figure 1a and b). The mean body weight loss was around 10% (Figure 1d). On gross pathology, a few dark red and raised areas were seen in the lungs of some animals and were consistent with mild pulmonary consolidation. Estimated areas of lung affected ranged from 0 to 10% (Figure 2a) between 0.5 and 14 dpi. The relative lung weight was comparable to that of non-infected ferrets (Figure 2b). There was a mild splenomegaly from 0.5 to 14 dpi (data not shown). The trachea-bronchial lymph nodes were slightly enlarged between 0.5 and 4 dpi, with a peak on 2 dpi (Table 1).

**Table 1:** Survival and clinical signs in ferrets inoculated with different influenza viruses.

			Clinical signs per group: median (range)										
Virus	Days post inoculation	Percentage of survival^a^ (n of total)	Activity status (0–3)	Inappetence (0–1)	Sneezing (0–1)	Dyspnea (0–1)	Nasal discharge (0–1)	Conjunctival discharge (0–1)	Diarrhea (0–1)	Nervous symptoms (0–1) (n of total)^a^	Tracheo-bronchial lymph node scores (median and range)	Virus excretion rectal swab [number positive/total (log_10_ TCID_50_/ml)]^b^	Homologous VN antibody titers (median and range)
H3N2	0.5	100% (28/28)	0	0	0 (0–1)	0	0	0	0	0	0	0/4	<8
	1	100% (24/24)	0	0	0	0	0	0	0	0	1 (0–1)	0/4	<8
	2	100% (20/20)	0.2 (0–1)	0	1	0	0	0 (0–1)	0	0	1.5 (1–2)	0/4	<8
	3	100% (16/16)	0	0	1	0	0	0	0	0	0.5 (0–1)	0/4	<8
	4	100% (12/12)	0	0	1	0	0	0	0	0	0.5 (0–1)	0/4	<8
	7	100% (8/8)	0	0	1	0	0	0	0	0	0 (0–1)	1/4 (2.8)	397 (256–609)
	14	100% (4/4)	0	0	1	0	0	0	0	0	0	0/4	1 669 (1 024–3 444)
pH1N1	0.5	100% (28/28)	0	0	0	0	0	0	0	0	0	0/4	<8
	1	100% (24/24)	0	0	0	0	0	0	0	0	2 (1–2)	2/4 (2.8)	<8
	2	100% (20/20)	1	1	1	0	0	0	0	0	1.5 (0–2)	0/4	<8
	3	100% (16/16)	1	1	1	0	0	0	0	0	2 (0–2)	1/4 (1.0)	<8
	4	100% (12/12)	1	1	1	0	0	0	0	0	3	1/4 (1.0)	<8
	7	100% (8/8)	1	0	1	0	0	0	0	0	4	0/4	9 423 (4096–2 3170)
	14	100% (4/4)	0	0	1	0	0	0	0	0	3	0/4	2 1327 (1 3777–2 3912)
H5N1	0.5	100% (20/20)	0	0	0	0	0	0	0	0	0	0/4	<8
	1	100% (16/16)	1	0.5 (0–1)	0	0	0	0	0.8 (0–1)	0	1 (0–3)	0/4	<8
	2	50% (6/12)	2 (2–3)	1	0	0	0	0	0.3 (0–1)	1 (1/12)	0 (0–2)	0/4	<8
	3	50% (3/6)	2 (2–3)	1	0	1	0	0	0	1 (2/6)	0	0/4	<8
	4	100% (3/3)	2 (2–3)	1	0	1	0	0	0	1 (2/3)	2 (0–2)	0/4	<8
Control	0.5	100% (28/28)	0	0	0 (0–1)	0	0	0	0	0	0	0/4	<8
	1	100% (24/24)	0	0	0	0	0	0	0	0	0	0/4	<8
	2	100% (20/20)	0	0	0	0	0	0	0	0	0 (0–1)	0/4	<8
	3	100% (16/16)	0	0	0	0	0	0	0	0	0	0/4	<8
	4	100% (12/12)	0	0	0	0	0	0	0	0	0 (0–2)	0/4	<8
	7	100% (8/8)	0	0	0	0	0	0	0	0	1 (0–2)	0/4	<8
	14	100% (4/4)	0	0	0	0	0	0	0	0	0 (0–2)	0/4	<8

Data also are provided for enlargement of the tracheo-bronchial lymph nodes, virus excretion from the rectum, and virus neutralization antibody titers in the serum.

a Scores are based on individual animals.

b The cut off values are <0.8 log_10_ TCID_50_/ml).

#### Histopathology and virus antigen expression

**Figure 3:**
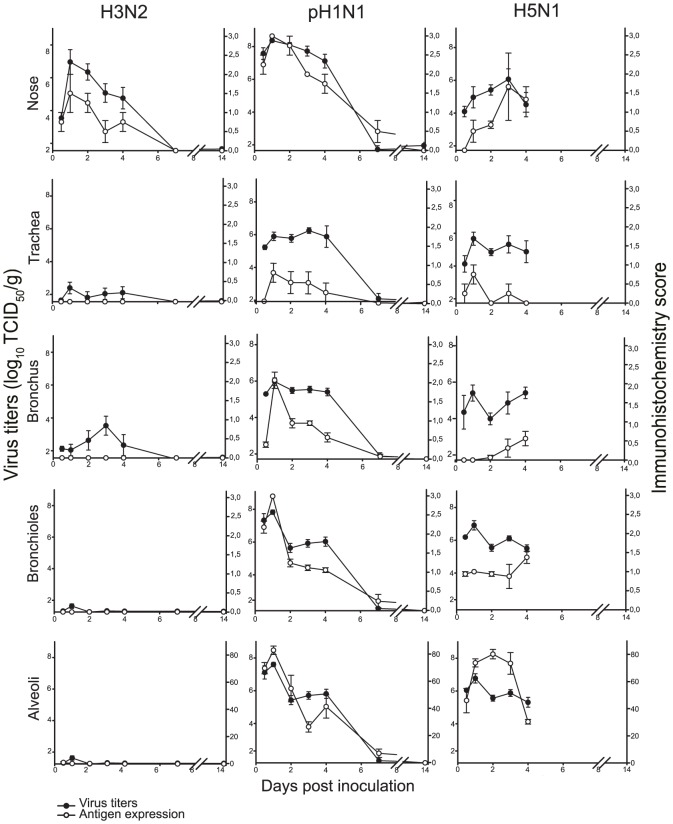
Virus distribution in the respiratory tracts of ferrets inoculated with different influenza viruses. The data are depicted as median values and standard errors of the mean. Virus titers and antigen expression as demonstrated by immunohistochemistry scores are comparable for ferrets inoculated with H3N2 and pH1N1. Ferrets inoculated with H5N1 have highest antigen expression in the alveoli while virus titers are comparable for all parts of the respiratory tract, suggesting that most virus originated from the lower respiratory tract. The average cut-off values for the respiratory tissues are: nose 1.6 (range 1.3–1.8), trachea 1.6 (1.4–1.9), bronchus 1.6 (range 1.3–2.0) and lung 1.3 (range 0.9–1.4) log_10_ TCID_50_. Immunohistochemistry scores in nose, trachea, bronchi, and bronchioles are scored from 0 to 3, and those in the alveoli are scored as a percentage.

**Figure 4:**
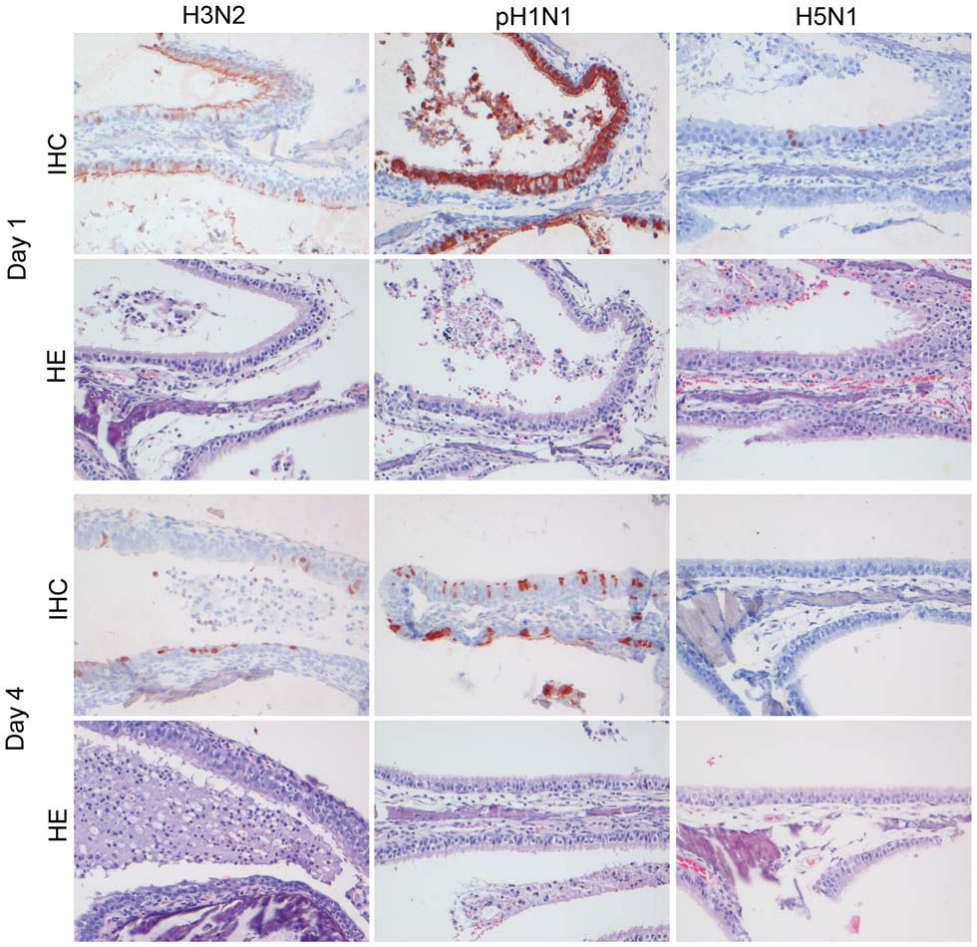
Antigen expression and histopathology of nasal respiratory epithelium of ferrets inoculated with different influenza viruses. There is high antigen expression with associated lesions characterized by neutrophilic rhinitis in the nasal respiratory epithelium of ferrets inoculated with H3N2 and pH1N1 on 1 dpi and lower expression on 4 dpi, while for H5N1 there is only occasional expression on 1 dpi. HE and immunoperoxidase counterstained with hematoxylin, 200×.

**Figure 5:**
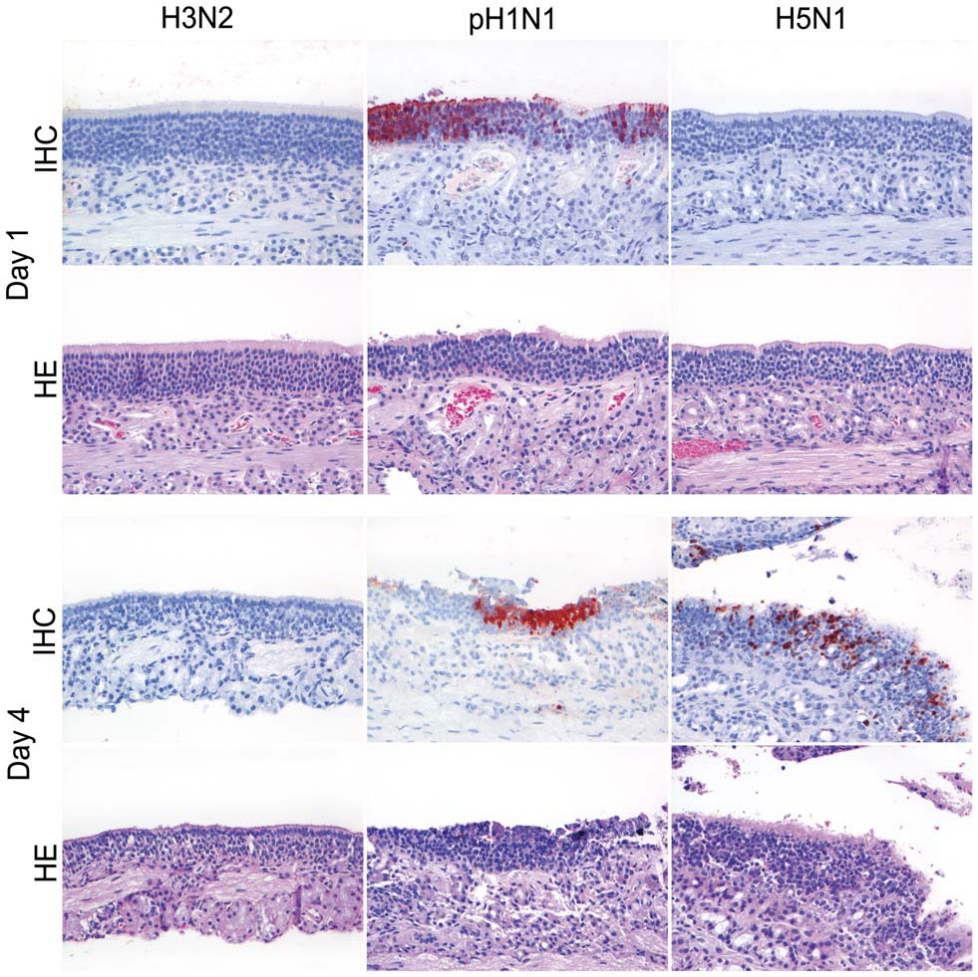
Antigen expression and histopathology of nasal olfactory epithelium of ferrets inoculated with different influenza viruses. There is high antigen expression with associated lesions characterized by neutrophilic rhinitis in the olfactory epithelium of ferrets inoculated with pH1N1 on 1 and 4 dpi, and for H5N1 only on 4 dpi. There were no antigen expression and no lesions for H3N2. HE and immunoperoxidase counterstained with hematoxylin, 200×.

**Figure 6:**
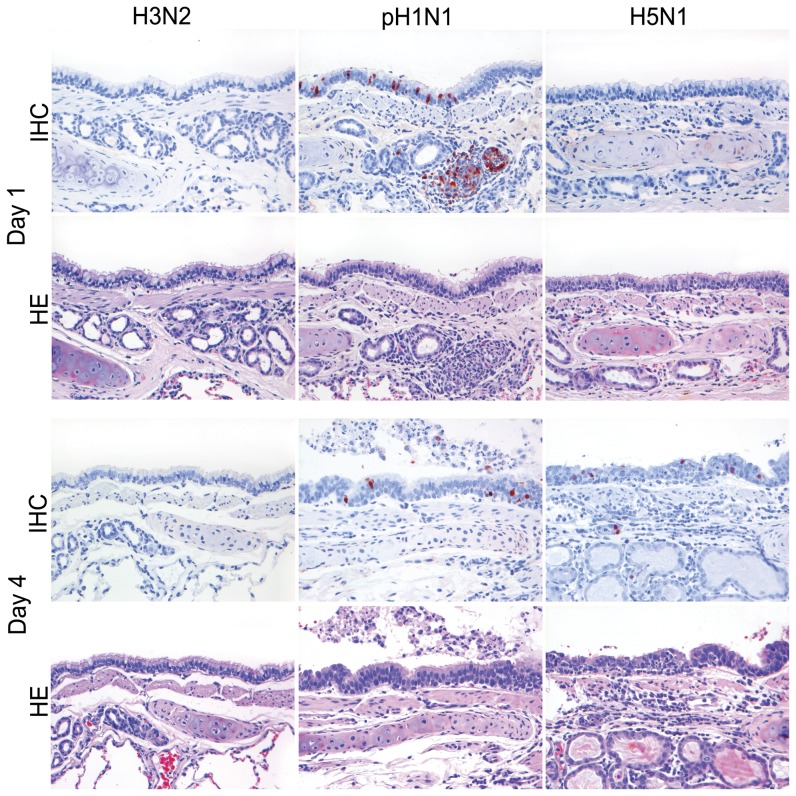
Antigen expression and histopathology of bronchial epithelium of ferrets inoculated with different influenza viruses. There is moderate antigen expression with associated lesions characterized by bronchitis in bronchial and glandular epithelium of ferrets inoculated with pH1N1 on 1 and 4 dpi, and for H5N1 only on 4 dpi. There were no antigen expression and no lesions for H3N2. HE and immunoperoxidase counterstained with hematoxylin, 200×.

**Figure 7:**
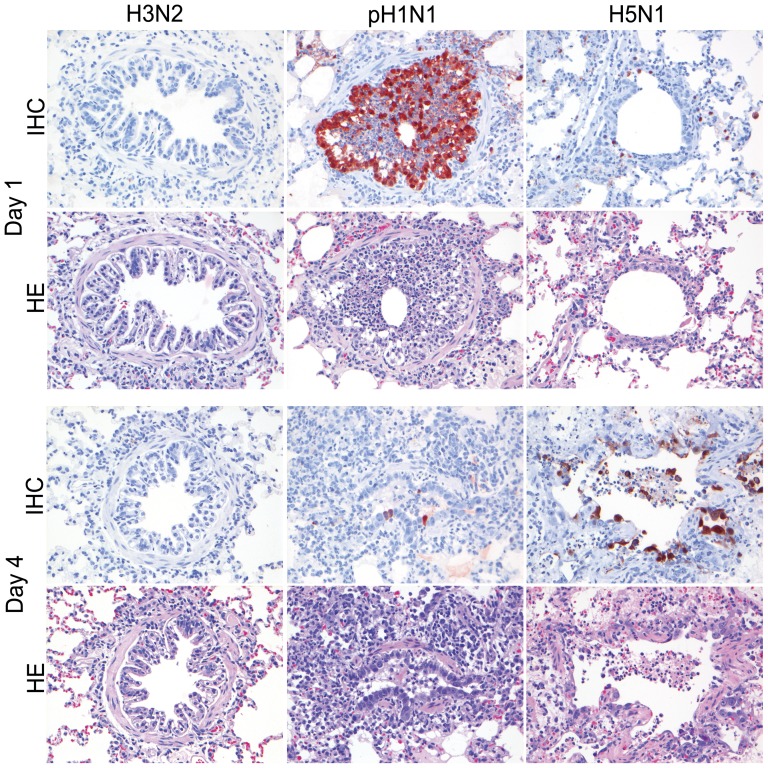
Antigen expression and histopathology of bronchiolar epithelium of ferrets inoculated with different influenza viruses. There is high antigen expression with associated lesions characterized by bronchiolitis in bronchiolar epithelium of ferrets inoculated with pH1N1 on 1 dpi that was low on 4 dpi, consistent with severe loss of bronchiolar epithelium, and for H5N1 there was no expression on 1 dpi and moderate antigen expression with associated lesions on 4 dpi. There were no antigen expression and no lesions for H3N2. HE and immunoperoxidase counterstained with hematoxylin, 200×.

**Figure 8:**
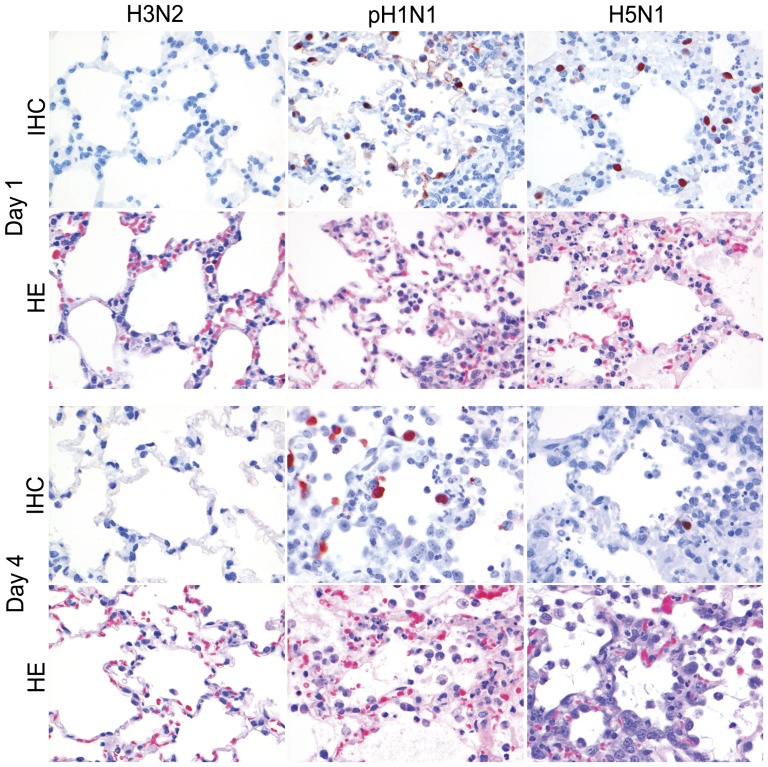
Antigen expression and histopathology of alveolar epithelium of ferrets inoculated with different influenza viruses. There was moderate to high antigen expression for pH1N1 and H5N1 on 1 and 4 dpi with associated lesions characterized by severe DAD and no expression and lesions for H3N2. HE and immunoperoxidase counterstained with hematoxylin, 400×.

By histopathology, there was a mild to severe multifocal rhinitis with necrosis of the epithelium and mild multifocal tracheitis. In the lungs, there was a mild multifocal bronchitis and bronchiolitis with intra-epithelial neutrophils, mild peribronchiolar and perivascular cuffing, mild broncho-adenitis, and mild multifocal alveolitis with mild intra-epithelial infiltration of neutrophils with mild epithelial necrosis and mild edema in the alveolar lumina. By immunohistochemistry, influenza virus antigen expression was visible as diffuse to granular red staining, which usually was stronger in the nucleus than in the cytoplasm (Figures 3, 4, 5, 6, 7, 8). Antigen expression in the nose was present from 0.5 to 4 dpi, while no antigen expression was seen in the tracheal, bronchial, bronchiolar, and tracheo-bronchial glandular epithelium at any time point, and little antigen expression in few type II pneumocytes in the alveoli. Changes in the histological lesions and antigen expression over time are described in the supporting information (Supporting Information S1).

Semiquantitative histological scoring (Table 2) showed that the extent and severity of the alveolar lesions were comparable for all days of sampling with no obvious changes. No expression of virus antigen was seen in extra-respiratory tissues on any days. By digital scoring, there was on average 63 to 73% of air in the pulmonary tissue (Table 3). Additionally, the percentage of antigen-expressing tissue and counts of virus antigen expression were absent and negligible, respectively (Table 3).

**Table 2:** Histopathology scores in ferrets inoculated with different influenza viruses.

		Histopathology score: median (range)									
Virus	Days post inoculation	Severity of alveolitis and alveolar damage (0–3)	Extent of alveolitis and alveolar damage (0–3)	Presence of alveolar edema (0–1)	Presence of alveolar hemorrhage (0–1)	Presence of hyperplasia and hypertrophy (0–1)	Presence of bronchitis and bronchiolitis (0–3)	Presence of perivascular and peribronchiolar cuffing (0–3)	Presence of tracheitis (0–3)	Presence of rhinitis (0–3)	Number of neutrophils in the alveolar wall in 20 hpf per animal (median and range)
H3N2	0.5	1.0 (1–1.75)	1.4 (1.25–2.25)	0 (0–0.25)	0	0	0.6 (0.15–1.5)	0.9 (0.5–1.5)	1.0 (0–2)	0	70.0 (52–106)
	1	0.9 (0.75–1)	1.4 (1–1.75)	0	0	0	0.4 (0–0.75)	0.9 (0–1.75)	0.5 (0–2)	0 (0–1)	71.0 (28–148)
	2	1.0	1.8 (1–2.25)	0	0	0	0.1 (0–0.25)	0.8 (0–1.25)	0 (0–1)	0	102.5 (86–124)
	3	0.8 (0.75–1)	1.6 (1–2.25)	0 (0–0.25)	0	0	0.1 (0–0.25)	0.6 (0–0.75)	0	1.5 (0–3)	79.0 (53–99)
	4	1.0 (0.75–1.25)	2.3 (1.25–2.50)	0 (0–0.25)	0	0	0.1 (0–0.5)	0.6 (0.25–0.75)	0 (0–1)	2.5 (2–3)	102.0 (63–143)
	7	1.0 (0.75–1.25)	1.8 (1–2)	0	0	0	0.3 (0–1)	1.1 (1–1.5)	1.0 (0–2)	2.5 (1–3)	75.0 (51–82)
	14	0.8	0.9 (1–1.50)	0	0	0	0 (0–0.25)	0.1 (0.25–0.75)	0	1.0 (0–2)	55.0 (22–61)
pH1N1	0.5	1.3 (1.25–1.75)	1.9 (1.5–3)	0	0	0	0.5 (0.25–1.75)	0.5 (0.15–1.5)	0 (0–2)	0 (0–1)	57.5 (28–93)
	1	2.5 (1.5–2.75)	2.6 (2.50–3)	0.6 (0–1)	0.1 (0–0.25)	0.1 (0–0.25)	2.9 (2.75–3)	1.8 (1.5–2)	0 (0–1)	2.0 (1–3)	124.5 (98–256)
	2	2.5 (2–2.75)	2.4 (2.25–2.5)	0.6 (0.25–1)	0.3 (0.25–0.5)	0.3 (0.25–0.5)	3.0 (2.25–3)	1.8 (1.5–2)	1.0	1.0 (0–2)	104.5 (72–145)
	3	2.5 (2.25–2.75)	2.8 (2–3)	0.8 (0.5–1)	0.4 (0.25–0.5)	0.4 (0.25–0.5)	2.6 (2.25–2.75)	1.8 (1.5–2)	1.0 (0–2)	2.5 (1–3)	128.0 (104–142)
	4	2.8 (2–3)	2.6 (2.5–2.75)	1.0 (0.75–1)	0.6 (0.25–0.75)	0.6 (0.25–0.75)	2.6 (2–2.75)	2.0 (1.5–2)	0.5 (0–1)	2.5 (0–3)	99.0 (78–144)
	7	2.6 (2.25–3)	2.6 (2.25–3)	0.9 (0.75–1)	0.8	0.8	3.0 (2.75–3)	2.4 (2.25–2.75)	2.4 (2.25–2.75)	3.0 (2–3)	120.0 (104–149)
	14	1.8 (1.75–2)	2.1 (1.75–2.5)	0	0	0	0.8 (1–1.5)	1.5 (1.5–2)	1.0 (0–2)	1.0 (1–2)	56.0 (17–63)
H5N1	0.5	2.1 (1.75–2.75)	2.4 (2–3)	0.1 (0–0.75)	0 (0.75-0)	0 (0–0.75)	2.1 (1.75–2.75)	2.0 (1.75–2.0)	1.5 (1–3)	1.5 (1–2)	70.5 (45–110)
	1	2.6 (2.25–2.75)	2.4 (2–2.5)	0.9 (0.5–1)	0.6 (0.25–1)	0.6 (0.25–1)	2.8 (2–2.75)	2.0 (1.75–2)	1.5 (0–3)	0.5 (0–2)	170.0 (88–211)
	2	2.8 (2.75–3)	2.8 (2.5–3)	1.0	0.8 (0.75–1)	0.8 (0.75–1)	2.9 (1.75–3)	1.9 (0–2)	2.0 (0–2)	0.5 (0–2)	132.5 (77–190)
	3	3.0 (2.5–3)	3.0 (2.25–3)	1.0 (0.75–1)	1.0 (0.75–1)	1.0 (0.75–1)	2.5 (2.25–3)	2.0 (1–2)	1.0	1.0	135.0 (46–190)
	4	3.0 (2.75–3)	3.0	1.0	1.0	1.0	2.8 (2.25–2.75)	1.3 (1.25–1.5)	3.0 (2–3)	1.0 (0–1)	105.0 (105–125)
Control	0.5	1.4 (1–1.75)	1.9 (1.75–2.5)	0.1 (0–0.25)	0.1 (0–0.25)	0.1 (0–0.25)	0.9 (0.25–1.25)	1.1 (1–2)	1.5 (0–2)	0.5 (0–2)	58.5 (42–82)
	1	1.0 (0.75–2.25)	1.4 (0.75–2.25)	0 (0–0.75)	0	0 (0–0.25)	1.3 (0.5–2)	1.4 (1.25–1.75)	1.5 (0–2)	1.0 (0–2)	51.0 (37–94)
	2	1.9 (1–2)	2.4 (1.25–3)	0.1 (0–0.25)	0	0 (0–0.25)	1.1 (0.25–1.75)	1.6 (0.75–2)	1.5 (0–2)	1.5 (0–2)	45.0 (24–91)
	3	1.4 (1.25–2)	2.0 (2–2.25)	0	0	0	1.1 (0.75–1.25)	1.9 (1.5–2)	1.0 (0–2)	1.0 (0–1)	41.0 (27–107)
	4	1.1 (0.75–1.5)	1.6 (1.25–2.25)	0	0	0	0.8 (0.5–1.25)	1.5 (0.75–1.75)	2.5 (2–3)	1.5 (1–2)	62.5 (47–97)
	7	1.1 (1–1.5)	2.0 (1–2.25)	0	0	0	0.8	1.1 (1–1.5)	2.5 (2–3)	1.5 (1–3)	123 (68–154)
	14	1.3 (1–1.5)	1.6 (1.25–2)	0	0	0	0.9 (0–1.25)	1.1 (0.75–1.75)	2.0 (2–3)	2.0 (0–2)	59.0 (32–81)
Negative control		0.25	0.25	0	0	0	0.25 (0–0.25)	0.25 (0–0.5)	0	0	(7–47)

**Table 3:** Digital histopathology scores in ferrets inoculated with different influenza viruses.

		Digital score: median (range)		
Virus	Days post inoculation	Percentage of air containing space in pulmonary tissue	Percentage of antigen expression	Number of antigen expression counts
H3N2	0.5	66.6 (49.4–75.9)	0	1 (0–2)
	1	65.1 (61.1–69.2)	0	1 (0–1)
	2	62.7 (58.0–72.1)	0	0 (0–1)
	3	66.6 (63.4–73.7)	0	1 (0–2)
	4	67.4 (46.2–70.4)	0	0 (0–1)
	7	72.9 (68.9–86.7)	0	0.5 (0–4)
	14	66.5 (57.3–74.1)	0	0 (0–2)
pH1N1	0.5	64.7 (56.0–80.3)	0.63 (0.09–0.74)	87.5 (21–164)
	1	65.0 (48.4–69.0)	0.92 (0.557–1.49)	273.5 (115–522)
	2	63.0 (59.3–71.1)	0.10 (0.05–0.16)	30 (15–49)
	3	70.3 (63.8–78.0)	0.06 (0.01–0.08)	14 (1–19)
	4	77.1 (73.3–81.0)	0.03 (0–0.05)	5.5 (1–50)
	7	63.1 (45.4–78.0)	0 (0–0.01)	1 (1–2)
	14	65.4 (55.7–73.1)	0 (0–0.1)	0.5 (0–6)
H5N1	0.5	58.3 (56.5–72.7)	0.06 (0.12–0.78)	24 (4–264)
	1	48.9 (45.8–51.6)	0.50 (0.29–0.57)	265.5 (174–282)
	2	44.0 (38.8–58.3)	0.20 (0.04–0.31)	114 (24–148)
	3	44.2 (39.0–56.9)	0.11 (0.09–0.20)	59 (43–73)
	4	38.9 (27.0–51.2)	0.04 (0.02–0.08)	19 (13–39)
Control	0.5	63.3 (48.7–74.8)	0	0
	1	66.4 (56.4–74.8)	0	0 (0–2)
	2	64.1 (53.4–70.4)	0	0
	3	57.8 (46.4–71.2)	0	0
	4	53.7 (42.1–67.1)	0	0.5 (0–1)
	7	63.4 (56.1–67.9)	0	0.5 (0–1)
	14	68.4 (61.1–77.4)	0	0 (0–1)
Negative control		81.1 (78.6–82.8)	0 (0–0.01)*	1.5 (0–2)*

* Antigen expression counts in the negative control animals are considered false positive counts due to the incapacity of the program to determine artifact staining.

#### Virology of tissues

Comparable with the pattern of antigen expression in the respiratory tissues, high virus titers were seen in the nasal concha from 0.5 to 4 dpi with a peak on 1 dpi (Figure 3). Low virus titers were present in the trachea, bronchi, lungs, tracheo-bronchial lymph nodes and tonsil (Figure 3 and Table 4). All other tissues did not contain replication competent virus.

**Table 4:** Virus isolation from tissues in ferrets inoculated with different influenza viruses.

		Presence of virus (number of animals positive/total number of animals) and virus titer (range log_10_ TCID_50_/gram tissue)											
Virus	Days post inoculation	Tracheo-bronchial lymph node	Tonsil	Olfactory bulb	Cerebrum	Cerebellum	Liver	Spleen	Heart	Kidney	Adrenal gland	Pancreas	Jejunum
H3N2	0.5	0/4 (2.0–2.2)	0/4	1/4 (2.9)	0/4	0/4	0/4	0/4	0/4	0/4	0/4	0/4	0/4
	1	0/4 (2.0–2.4)	4/4 (2.7–3.8)	1/4 (1.7)	0/4	0/4	0/4	0/4	0/4	0/4	0/4	0/4	0/4
	2	0/4 (1.6–2.3)	2/4 (2.5–3.3)	1/4 (1.8)	0/4	0/4	0/4	0/4	0/4	0/4	0/4	0/4	0/4
	3	1/4 (1.8)	1/4 (2.8)	1/4 (1.8)	0/4	0/4	0/4	0/4	0/4	0/4	0/4	0/4	0/4
	4	0/4	1/4 (2.2)	0/4	0/4	0/4	0/4	0/4	0/4	0/4	0/4	0/4	0/4
	7	0/4	0/4	0/4	0/4	0/4	0/4	0/4	0/4	0/4	0/4	0/4	0/4
	14	0/4	0/4	0/4	0/4	0/4	0/4	0/4	0/4	0/4	0/4	0/4	0/4
pH1N1	0.5	2/4 (2.0–2.5)	4/4 (3.3–5.5)	3/4 (1.9–3.3)	3/4 (1.3–1.9)	1/4 (2.2)	0/4	0/4	0/4	0/4	0/4	0/4	0/4
	1	4/4 (3.8–4.5)	4/4 (4.8–6.2)	4/4 (1.6–5.4)	2/4 (2.0–3.2)	3/4 (1.6–2.9)	1/4 (1.4)	0/4	2/4 (1.6–2.6)	0/4	0/4	0/4	0/4
	2	4/4 (2.0–3.3)	4/4 (2.9–4.4)	4/4 (2.2–5.7)	3/4 (1.7–3.7)	2/4 (1.9–3.6)	0/4	0/4	1/4 (3.0)	0/4	0/4	0/4	0/4
	3	4/4 (2.0–2.8)	4/4 (3.7–5.3)	4/4 (2.0–4.5)	4/4 (1.2–3.8)	3/4 (1.3–4.1)	0/4	0/4	2/4 (1.7–2.4)	0/4	0/4	0/4	0/4
	4	4/4 (1.6–2.2)	3/4 (4.3–5.1)	4/4 (2.1–5.7)	3/4 (1.6–4.1)	3/4 (1.2–3.7)	0/4	0/4	2/4 (1.2–2.2)	0/4	0/4	0/4	0/4
	7	0/4	0/4	2/4 (1.7–1.8)	0/4	1/4 (1.3)	0/4	0/4	0/4	0/4	0/4	0/4	0/4
	14	0/4	0/4	0/4	0/4	0/4	0/4	0/4	0/4	0/4	0/4	0/4	0/4
H5N1	0.5	4/4 (3.4–4.7)	3/4 (2.2–4.3)	1/4 (1.9)	1/4 (0.9)	0/4	0/3^*^	0/4	3/4 (0.9–1.2)	0/4	0/4	0/1^a^^*^	0/1^a^^*^
	1	4/4 (3.1–6.2)	4/4 (3.8–4.5)	2/4 (2.3–2.7)	2/4 (1.6–1.8)	2/4 (1.4–1.5)	2/4 (1.0–1.8)	4/4 (1.5–2.5)	2/4 (1.0–1.2)	3/4 (1.3–2.1)	1/4 (1.7)	nd	1/4 (1.6)
	2	6/6 (2.7–7.2)	6/6 (3.4–4.4)	5/6 (1.5–3.9)	4/6 (1.2–3.5)	0/6	3/6 (1.5–3.6)	5/6 (1.9–3.4)	3/6 (1.1–1.8)	3/6 (1.0–1.6)	3/6 (1.4–2.6)	nd	3/6 (4.2–5.2)
	3	3/3 (5.2–6.7)	3/3 (4.4–6.1)	2/3 (3.5–5.8)	1/4 (2.5)	2/3 (3.1–3.2)	3/3 (1.1–1.8)	3/3 (3.9–4.5)	2/3 (1.3–1.6)	1/3 (1.5)	2/3 (2.4–2.9)	nd	1/3 (4.5)
	4	3/3 (4.9–6.3)	3/3 (3.9–5.4)	2/3 (2.5–3.3)	3/4 (1.0–3.8)	2/3 (1.4–2.0)	3/3 (1.0–2.1)	3/3 (2.1–5.2)	3/3 (1.4–3.2)	2/3 (1.3–1.4)	2/3 (1.4–2.6)	nd	0/3
Cut off (Range)^b^		1.1–2.4	1.7–2.6	1.3–2.5	0.9–1.9	1.0–1.8	0.9–1.6	0.9–1.6	0.3–1.4	1.1–1.7	1.3–1.9	1.3–1.8	1.1–1.6

a Virus titers of the other animals in the group could not be determined.

b The range of the cut off values is the range of the cut off values of the individual animals.

nd, not determined.

#### Hematology and comparison of leucocytes in blood and alveolar lumina

**Figure 9:**
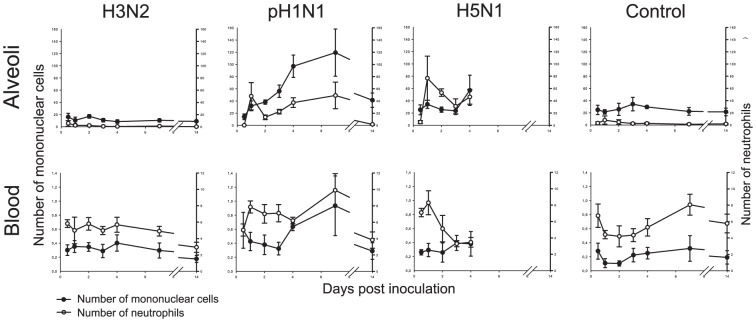
Leucocyte counts in alveoli and blood of ferrets inoculated with different influenza viruses. The number of leucocytes in the alveolar lumina (scores) and blood (×10^9^/L) are depicted as median values and standard errors of the mean. The number of mononuclear cells and neutrophils in the alveolar lumina and blood follow the same pattern for H3N2 and pH1N1. The increase of the number of mononuclear cells in the alveoli and decrease in the blood for pH1N1 demonstrates the demand in the alveoli. For pH1N1 there is a peak of neutrophils in the alveoli on 0.5 dpi followed by a dip and a response of myelopoiesis in the blood. For H5N1 the number of mononuclear cells is not elevated in the alveoli and blood, and the high demand for neutrophils in the alveoli is not followed by higher release of neutrophils in the blood. In negative control animals (non-inoculated) the median number (range) of mononuclear cells is 0.30 10^9^/L (0.13–0.37) in the blood and 17 (5–22) in the alveolar lumina, for neutrophils 4.33 (3.07–7.28) in the blood and 0.5 (0–2) in the alveolar lumina.

Total leucocyte counts after infection were slightly increased on all days except for 14 dpi compared to those in negative control animals (Table 5). In blood, there was a slight increase in the number of mononuclear cells on 1 dpi and of neutrophils on 2 dpi, followed by a mild decrease up to 4 dpi after which again a small increase of both was observed (Figure 9). In the alveolar lumina, the number of mononuclear cells was only slightly increased on 2 dpi (Figure 8).

**Table 5:** Leucocyte concentrations in the peripheral blood of ferrets inoculated with different influenza viruses.

		Concentration of cells (10^9^/L)						
Virus	Days post inoculation	Total leucocytes	Lymphocytes	Blastocytes	Rod-shaped neutrophils	Eosinophils	Basophils	Normoblasts
H3N2	0.5	9.6 (7.2–10.0)	2.59 (2.23–3.60)	0	0.04 (0–0.09)	0.19 (0–0.18)	0 (0–0.07)	0
	1	9.3 (4.2–14.1)	3.76 (2.14–4.51)	0	0	0.27 (0.08–0.55)	0 (0–0.08)	0
	2	8.3 (7.0–10.5)	2.18 (1.61–2.38)	0	0.04 (0–0.11)	0.27 (0–0.41)	0	0 (0–0.08)
	3	8.9 (7.6–9.3)	2.97 (1.95–3.59)	0	0 (0–0.09)	0.40 (0.09–0.53)	0.09 (0–0.34)	0
	4	9.1 (6.3–11.0)	2.23 (1.89–3.46)	0	0	0.30 (0.22–0.44)	0 (0–0.07)	0
	7	7.9 (5.8–9.5)	2.24 (2.03–2.61)	0	0 (0–0.09)	0.23 (0–0.17)	0.04 (0–0.10)	0
	14	5.8 (3.0–6.7)	1.95 (1.16–2.72)	0	0	0.26 (0–0.46)	0 (0–0.06)	0
pH1N1	0.5	9.9 (6.2–10.9)	2.95 (2.67–4.46)	0	0 (0–0.20)	0.30 (0.12–0.33)	0	0
	1	9.1 (8.6–12.1)	1.00 (0.73–1.29)	0	0.09 (0–0.36)	0.18 (0.17–0.46)	0 (0–0.12)	0
	2	9.4 (6.7–11.4)	1.66 (1.46–1.84)	0	0 (0–0.11)	0.03 (0–0.34)	0 (0–0.07)	0
	3	10.8 (7.9–11.2)	2.25 (1.56–4.31)	0	0 (0–0.11)	0.10 (0–0.21)	0 (0–0.11)	0
	4	8.5 (7.3–9.0)	1.31 (0.79–1.68)	0	0.04 (0–0.36)	0.04 (0–0.18)	0 (0–0.09)	0
	7	11.5 (10.7–20.7)	2.19 (1.44–4.17)	0	0	0 (0–0.62)	0.11 (0–0.22)	0
	14	9.0 (6.6–10.6)	4.57 (2.90–5.86)	0	0.05 (0–0.13)	0.15 (0–0.19)	0	0
H5N1	0.5	10.5 (8.5–12.4)	2.46 (1.32–4.34)	0	0 (0–0.11)	0.25 (0.10–1.10)	0 (0–0.12)	0
	1	8.0 (6.6–14.1)	0.27 (0.07–0.99)	0	0.19 (0–0.34)	0 (0–0.07)	0	0
	2	5.4 (2.6–13.5)	0.47 (0.21–0.81)	0	0.06 (0.03–0.49)	0.03 (0–0.06)	0	0
	3	4.6 (4.1–5.0)	0.72 (0.70–0.74)	0	0.14 (0.12–0.15)	0.02 (0–0.04)	0	0
	4	5.8 (3.3–6.1)	0.73 (0.73–0.99)	0	0.46 (0.23–0.49)	0 (0–0.06)	0	0
Control	0.5	11.2 (9.0–19.0)	5.41 (2.97–6.84)	0	0 (0–0.19)	0.27 (0.09–0.42)	0.05 (0–0.12)	0
	1	9.1 (7.9–11.9)	3.97 (3.16–7.73)	0	0	0.16 (0.10–0.48)	0 (0–0.08)	0
	2	7.8 (5.9–11.8)	3.71 (2.89–5.09)	0	0 (0–0.12)	0.11 (0–0.24)	0	0
	3	10.1 (6.5–12.1)	4.07 (3.45–8.23)	0	0	0.11 (0–0.35)	0 (0–0.07)	0
	4	9.1 (5.3–15.2)	3.35 (2.06–6.38)	0	0 (0–0.05)	0.32 (0.20–0.46)	0	0
	7	13.6 (7.5–14.0)	3.15 (2.03–6.07)	0	0	0.18 (0.13–0.28)	0	0
	14	11.7 (6.1–15.6)	5.94 (2.20–6.71)	0	0 (0–0.16)	0.05 (0–0.16)	0	0
Negative control		7.6 (6.4–9.1)	2.86 (1.45–3.28)	0	0	0.03 (0–0.08)	0	0

#### Generalized linear model of viral excretion

We used generalized linear models (GLM) to define possible predictors of viral excretion, based on viral production and damage scores in upper (nose and trachea) and deeper regions of the respiratory tract (bronchi, bronchioles and alveoli). These models were used to determine significant linear relationships between these scores and viral excretion (as measured by viral titers in nose and throat swabs; see methods). The final GLM predicting excretion of H3N2 included only viral production in the upper regions of the respiratory tract as a significant predictor (LR χ^2^ = 16.8, df = 1, *P*<0.0001). Excretion of H3N2 was a positive linear function of viral production in the upper regions (Table 6). As such, viral excretion of H3N2 could be directly estimated based on viral production scores in the nose and trachea, strongly suggesting that these regions were the main sources of excreted virus.

**Table 6:** Parameter-estimates of generalized linear models predicting viral excretion of ferrets inoculated with different influenza viruses.

Virus	Predictor	B coefficient	Standard error	Wald χ^2^	*P*
H3N2	Intercept	1.605	0.34	22.3	<0.0001
	Viral production in URRT	0.383	0.08	22.3	<0.0001
pH1N1	Intercept	0.325	0.25	1.7	0.187
	Viral production in URRT	0.508	0.04	201.7	<0.0001
H5N1	Intercept	27.830	10.06	7.6	0.006
	Viral production in DRRT	−2.827	1.19	5.6	0.018
	Damage in DRRT	−3.887	1.72	5.1	0.024
	Viral production in DRRT×Damage in DRRT	0.456	0.20	5.0	0.025
	Damage in URRT	−0.687	0.20	11.8	0.001
	Damage in URRT×Viral production in URRT	0.046	0.01	9.2	0.002
	Damage in URRT×Viral production in DRRT	0.049	0.02	4.6	0.031

URRT: upper regions of the respiratory tract (nose and trachea); DRRT: deeper regions of the respiratory tract (bronchi, bronchioles and alveoli).

### Pandemic H1N1

#### Clinical data and gross pathology

The survival rate was 100%. Clinical signs per group were observed from 2 to 14 dpi (Table 1). The activity status varied over the different time points with the highest score (status 3) seen in one animal between 5 and 8 dpi. Dyspnea was seen only in this animal. Inappetence, sneezing and nasal discharge was seen in all animals. Virus excretion was shown in nasal and pharyngeal swabs with a peak on 1 dpi and higher values in the nasal swabs. The virus titers for the pharyngeal swabs were comparable to those of the animals inoculated with H3N2 (Figure 1b). An increase in body temperature was seen with a peak on 1 dpi (Figure 1c). The mean body weight loss was up to 15% on 7 dpi (Figure 1d). By gross pathology, multifocal pulmonary consolidation was seen on 0.5 dpi with grey-red raised, and slightly firmer than normal areas. On 1 dpi, the percentage of affected lung tissue was increased (Figure 2a). On 2 dpi the lesions were dark red and firmer with increased relative lung weight. On 14 dpi the percentage of affected lung tissue decreased again (Figure 2a). The relative lung weight was increased from 1 to 7 dpi and decreased on 14 dpi (Figure 2b). The trachea-bronchial lymph nodes were enlarged from 0.5 to 7 dpi, with a peak on 7 dpi (Table 2). There was a mild splenomegaly from 0.5 to 14 dpi (data not shown).

#### Histopathology and virus antigen expression

**Figure 10:**
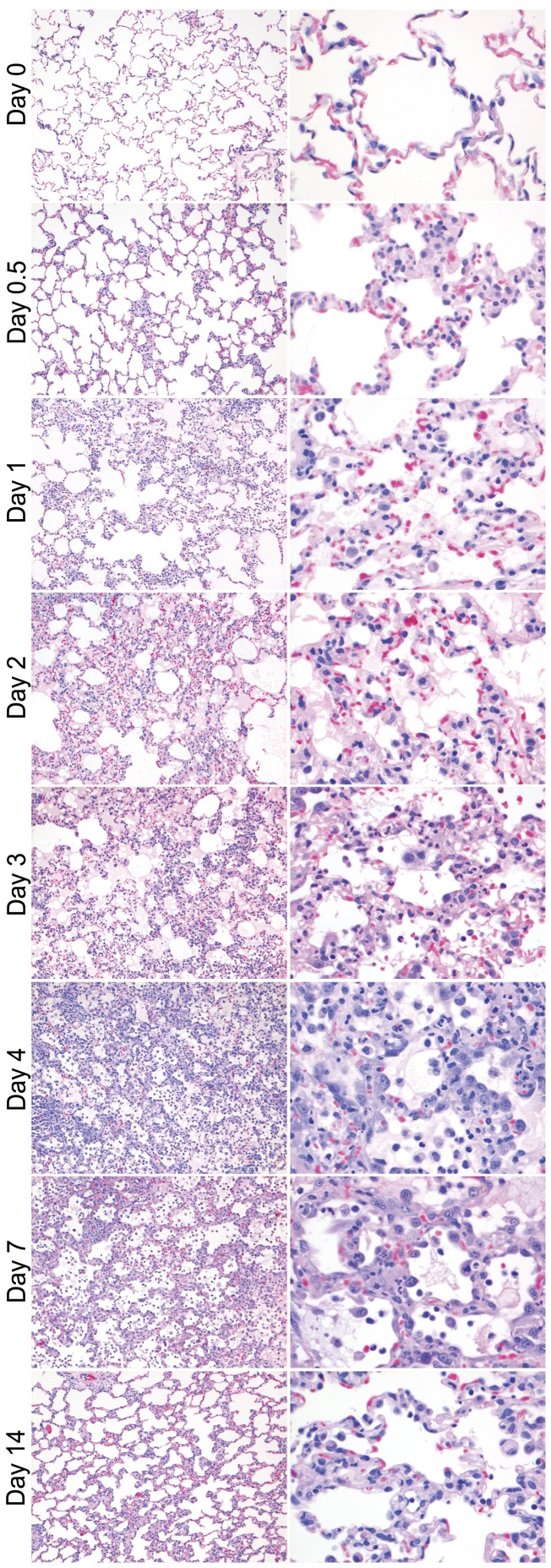
Histopathologic changes in the alveoli during time in ferrets inoculated with pH1N1. On 0.5 dpi there was mild DAD that was characterized by neutrophils in the alveolar walls. On 1 dpi the alveolar walls were thickened with mild necrosis of the lining epithelium and increased number of neutrophils, in the lumina there were more mononuclear cells, little amount of edema fluid mixed with fibrin, hemorrhage and little cellular debris. On 2 dpi there was increased presence of necrosis, edema, hemorrhage and hyperplasia. On 3 dpi there were both new lesions with acute necrosis and old lesions with type II hyperplasia present, with increased amounts of edema and hemorrhage. On 4 dpi the type II hyperplasia and hypertrophy was more pronounced with increased amount of edema and high numbers of mononuclear cells. On 7 dpi there was severe epithelial hyperplasia and hypertrophy as well as intraluminal edema and hemorrhage. On 14 dpi the walls were mildly thickened. HE, 100× (first column), 400× (second column).

**Figure 11:**
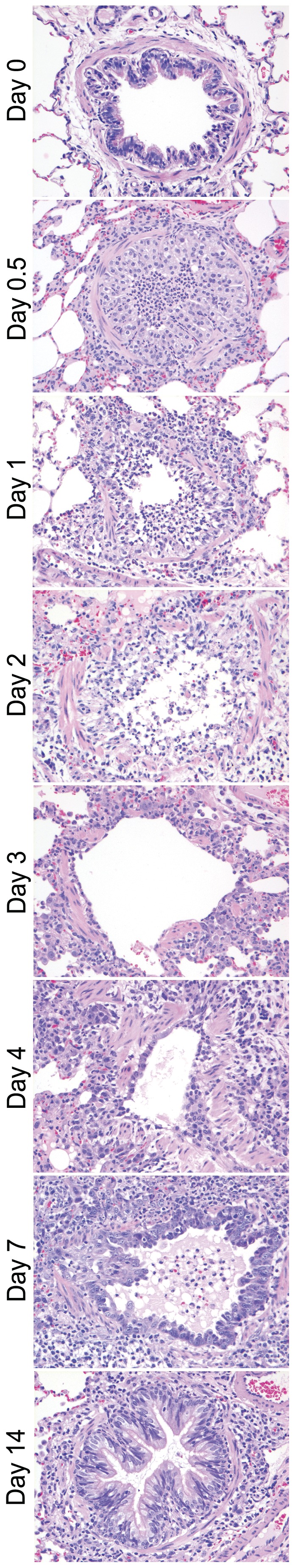
Histopathologic changes in the bronchioles during time in ferrets inoculated with pH1N1. On 0.5 dpi there was infiltration of neutrophils in the bronchiolar epithelium and lumen with mild peribronchiolar cuffing. On 1 dpi the number of neutrophils increased with necrosis of the epithelium, intraluminal cellular debris and peribronchiolar cuffing. On 2 dpi the necrosis was more severe than on 1 dpi. On 3 dpi there was denudation of the basement membrane and little regeneration of the bronchiolar epithelium with hyperplasia, peribronchiolar cuffing and occasionally cellular debris in the lumen. On 4 dpi there was increased epithelial hyperplasia with moderate cuffing. On 7 dpi the epithelium covered the basement membrane of the bronchiole with hypertrophic cells and moderate cuffing. On 14 dpi the epithelium was slightly hypertrophic with little cuffing. HE, 200×.

By histopathology, there was a mild to severe multifocal rhinitis with necrosis of the epithelium and mild to moderate multifocal tracheitis. In the lungs, there was a mild to severe multifocal bronchitis and bronchiolitis with intra-epithelial and intraluminal neutrophils, mild to severe necrosis of epithelium, mild to severe peribronchiolar and perivascular cuffing, mild to severe broncho-adenitis, and mild to severe multifocal alveolitis with infiltration of neutrophils in alveolar walls and lumina, mild to moderate epithelial necrosis and mild to moderate edema in the alveolar lumina with hypertrophy and hyperplasia of epithelium (Figures 10 and 11). The tracheo-bronchial lymph nodes and tonsils demonstrated lymphadenopathy, and in the palatine roof of two animals there was severe inflammation and necrosis of the sero-mucous glands.

Antigen expression in the nose was present from 0.5 to 7 dpi starting on 0.5 dpi in the respiratory epithelium and on 1 dpi in the olfactory epithelium. In the trachea there was antigen expression from 0.5 to 6 dpi and in the bronchi and bronchioles from 0.5 to 7 dpi with peak values on 1 dpi. Bronchial glandular epithelial cells expressed virus antigen with often more expression in glandular cells than in bronchial epithelial cells. In the alveoli there was antigen expression in type II pneumocytes and less in type I pneumocytes and alveolar macrophages from 0.5 to 7 dpi with highest values on 1 dpi. The high values decreased after 1 dpi consistent with the severe damage of the epithelium. The tracheo-bronchial lymph nodes and tonsils did not express virus antigen while glandular epithelial cells in the palatine roof of two animals did express antigen on 7 dpi. There was no virus antigen expression in extra-respiratory tissues. Changes in the histological lesions and antigen expression over time are described in the supporting information (Supporting information S1).

Digital scoring demonstrated that the amount of air-containing tissue was comparable for all days with averages between 63 and 77% (Table 3). When digital scoring for antigen expression was performed, antigen expression as well as the number of positive counts showed that virus antigen was clearly present on 0.5 dpi with peak scores on 1 dpi, albeit with high variation between the different animals (Table 3).

#### Virology

In the respiratory tissues, the changes in virus titers over time showed a comparable pattern to that of viral antigen expression, with high virus titers from 0.5 to 4 dpi (Figure 3). In the extra-respiratory tissues, virus was isolated from the olfactory bulb, cerebellum, cerebrum, and heart (Table 4).

#### Hematology and comparison of leucocytes in blood and alveolar lumina

Total leucocyte counts showed higher values on all time points when compared to those from negative control animals (Table 5). On 1 dpi, there was an increase of neutrophils in the blood and in the alveoli. On 2 dpi, there was a severe decrease in the number of neutrophils in the alveoli, but the number of neutrophils in the blood remained high. The number of mononuclear cells in the alveoli increased during the first 3 days while the number of mononuclear cells in the blood decreased. On 14 dpi, the numbers of neutrophils and mononuclear cells were decreased in both the blood and the alveoli.

#### Generalized linear model of viral excretion

Similarly to H3N2, the final GLM predicting excretion of pH1N1 included only viral production in the upper regions of the respiratory tract (nose and trachea) as a significant predictor (LR χ^2^ = 58.9, df = 1, *P*<0.0001). Excretion of pH1N1 was a positive linear function of viral production in the upper regions (Table 6).

### H5N1

#### Clinical data and gross pathology

On 2.5 dpi, one animal died and one animal was euthanized because of its moribund state. On 3 dpi, another animal died. From 2 to 4 dpi all animals developed severe clinical signs characterized by decreased activity, dyspnea, and inappetance (Table 1). Two animals showed nervous signs, characterized by ataxia, drifting to the right, walking into a corner and aggression. Virus was excreted in nasal and pharyngeal swabs, with lower virus titers than the pH1N1 and H3N2 groups during the first 3 days for the nose and the first 2 days for the pharynx (Figures 1a and b). In contrast to what was seen in the other virus groups, the nasal swabs showed lower titers than the pharyngeal swabs. This indicated lower replication in the nose, as was also suggested by the low levels of virus antigen expression and low virus titers in the nose (Figure 3). The increase in body temperature showed a peak on 1 dpi. At later time points, the average temperature decreased below baseline values due to the progressively moribund state of most of the ferrets (Figure 1c). The body weight loss increased during time and was comparable to that of the pH1N1 group (Figure 1d). By gross pathology, there was multifocal pulmonary consolidation on 0.5 dpi with dark red, raised, and firmer than normal areas affecting an average of 13% of the lung tissue (Figure 2a). On 1 dpi, there was fluid in the bronchi and lung parenchyma, and the percentage of affected lung tissue was increased. On 2 dpi, the percentage of affected lung tissue had increased dramatically to almost 60% (Figure 2a). The relative lung weights were increased on all time points and were much higher than in the other virus groups (Figure 2b). Tracheo-bronchial lymph nodes were enlarged starting on 0.5 dpi, and up to two times the normal size on 4 dpi. There was a mild splenomegaly on all days (data not shown).

#### Histopathology and virus antigen expression

By histopathology, there was a mild to moderate multifocal rhinitis with necrosis of the epithelium and mild multifocal tracheitis. In the lungs, there was a moderate multifocal bronchitis and bronchiolitis, with intra-epithelial and intraluminal neutrophils, mild to moderate epithelial necrosis, and mild peribronchiolar and perivascular cuffing; a mild to severe broncho-adenitis; and a severe multifocal to diffuse alveolitis, with severe epithelial necrosis, intra-epithelial and intraluminal infiltration of neutrophils, severe intraluminal edema, and hypertrophy and hyperplasia of epithelial cells (Figures 4, 5, 6, 7, 8). The tracheo-bronchial lymph nodes and tonsils demonstrated lymphadenopathy. In the extra-respiratory tissues there was lymphadenopathy in a sternal lymph node and there was hyperplasia and necrosis in the gut associated lymphoid tissue (GALT) of the jejunum and in the spleen of few animals.

In the nose there was antigen expression from 1 to 4 dpi with highest values on 2 and 3 dpi. Antigen expression in the trachea was present from 0.5 to 3 dpi, predominantly on 1 dpi. The bronchi demonstrated antigen from 2 to 4 dpi, and in the bronchioles from 0.5 to 4 dpi. Antigen expression in the alveoli was present in type II pneumocytes (Figure 8), and less in type I pneumocytes and alveolar macrophages. It was seen from 0.5 to 4 dpi with large percentages from 1 to 3 dpi. In the tracheo-bronchial lymph nodes there was antigen expression from 1 to 3 dpi and in the tonsils on 2 and 3 dpi in mononuclear cells. One animal had virus antigen expression in squamous stratified epithelium in the tip of the nose and one animal showed expression in endothelial cells in the pharynx. In the extra-respiratory tissues there was virus antigen expression in mononuclear cells in the GALT of the jejunum on 2 and 3 dpi and in the spleen on 4 dpi. No antigen expression was seen in other extra-respiratory tissues. Changes in the histological lesions and antigen expression over time are described in the supporting information (Supporting information S1).

Digital scoring demonstrated that the amount of air-containing tissue was lower than in the other virus groups, and decreased over time (Table 3). Digital scoring for antigen expression showed positive values on all time points with highest values on 1 dpi, but with high variation (Table 3).

#### Virology of tissues

In the nose, the virus titers were higher than expected based on virus antigen expression in the nose, indicating a different origin of the virus (Figure 3). In the lungs, virus titers on 1 dpi were higher than on the other days. This could be due to slower replication on 0.5 dpi and necrosis of virus-replicating cells on 4 dpi. In the extra-respiratory tissues, virus was isolated from the olfactory bulb, cerebrum, cerebellum, heart, spleen, liver, kidney, adrenal gland, pancreas, and jejunum in one or more animals from 0.5 to 4 dpi. The highest virus titers were seen on 2 dpi in olfactory bulb and jejunum, and on 3 and 4 dpi in the spleen (Table 4).

#### Hematology and comparison of leucocytes in blood and alveolar lumina

The total leucocyte counts were decreased from 1 to 4 dpi (Table 5). The number of neutrophils in the alveolar lumina was low on 0.5 dpi but increased strongly with a peak value on 1 dpi. However, in the blood, after a small peak on 1 dpi, there was a decrease of the number of neutrophils (Figure 9). The number of mononuclear cells in the alveolar lumina showed an increase on 4 dpi. This increase was later than in the pH1N1 group, while the number of mononuclear cells in the blood did not increase. The number of lymphocytes in the blood was decreased on all days (Table 5).

#### Generalized linear model of viral excretion

The final GLM predicting excretion of the H5N1 included 6 significant predictors (LR χ^2^ = 14.1, df = 6, *P* = 0.029): viral production and damage in the deeper regions of the respiratory tract (bronchi, bronchioles and alveoli; *P* = 0.018 and *P* = 0.024, respectively); their product (*P* = 0.025); damage in the upper regions of the respiratory tract (nose and trachea; *P* = 0.001); its product with viral production in these regions (*P* = 0.002); and its product with viral production in the deeper regions (*P* = 0.031). Excretion of H5N1 was a negative linear function of each of the three single predictors and a positive linear function of each of the three products. Coefficients are given in Table 6.

### Control group with inoculum

#### Clinical signs and gross pathology

No clinical signs were seen and there was a survival rate of 100%. The body weight loss in the control group increased slightly in time (Figure 1d). By gross pathology, the lungs had few dark red and slightly raised areas consistent with mild pulmonary consolidation. The area of affected lung tissue varied between 0 and 10% of the lung area (Figure 2a). The relative lung weight remained constant at around 0.6% on all time points (Figure 2b). Tracheo-bronchial lymph nodes were slightly enlarged (Table 1) and mild splenomegaly was present on all time points (data not shown).

#### Histopathology and virus antigen expression

By histopathology, there was mild multifocal rhinitis, tracheitis, bronchitis and bronchiolitis with few neutrophils, mild perivascular and peribronchiolar cuffing, mild broncho-adenitis, and mild focal alveolitis with infiltration of few neutrophils in the alveolar septa.

No virus antigen expression was seen in any of the tissues. Changes in the histological lesions over time are described in the supporting information (Supporting information S1). Digital scoring demonstrated that the amount of air-containing tissue was slightly lower on 3 and 4 dpi (Table 3).

#### Virology

All swabs and tissues were negative in virus titration for influenza virus.

#### Hematology and comparison of leucocytes in blood and alveolar lumina

No trends were observed in total leucocyte, segmented neutrophil, mononuclear cell and lymphocyte counts in the blood, or in comparison of leucocyte counts between blood and alveolar lumina (Figure 9 and Table 5).

## Discussion

Our time course experiments show how strongly the spatial and temporal dynamics of infection and associated lesions in the ferret respiratory tract differ between a seasonal human influenza virus (H3N2), a pandemic human influenza virus (pH1N1), and a zoonotic avian influenza virus (H5N1): H3N2 infection was restricted to the nose and peaked at 1 dpi; pH1N1 infection also peaked at 1 dpi, but occurred at similar levels throughout the respiratory tract; and H5N1 infection occurred predominantly in the alveoli, where it peaked for a longer period, from 1 to 3 dpi (Figure 3). The associated lesions followed the same spatial distribution as virus infection, but their severity peaked between 1 and 6 days later (Table 2): at 4 to 7 dpi for H3N2 and pH1N1, after which lesions decreased in severity by 14 dpi; and at 4 dpi for H5N1, when all H5N1-infected ferrets had either died or been euthanized on humane grounds. An important implication of these results is that location and timing of sample collection need to be chosen carefully in any comparative study of infection and pathology by different influenza viruses, otherwise the comparison will not be valid.

The main source of excreted virus likely differs between H3N2 and H5N1 infections, based on comparison of virus titers in nasal and pharyngeal swabs (Figure 1) with virus antigen expression in respiratory tract tissues (Figure 3). For H3N2, the temporal dynamics of nasal swab virus titers, pharyngeal swab virus titers, and virus antigen expression in the nose were comparable. Together with the lack of virus antigen expression in the lower respiratory tract, these results suggest that the nose is the main source of excreted H3N2. This is supported by the final GLM for H3N2 viral excretion, which included only viral production in the upper regions of the respiratory tract as significant predictor. In contrast, for H5N1, virus titers in both nasal swabs and pharyngeal swabs were already near peak levels at 1 dpi, while virus antigen expression in the nose remained relatively low until 3 dpi. Together with the higher levels of virus antigen expression in the trachea, bronchioles, and especially alveoli at 1 to 2 dpi, these results suggest that the lower respiratory tract is the main source of excreted H5N1, at least until 3 dpi. In fact, the final GLM for H5N1 viral excretion suggests that viral production and damage in the deeper regions of the respiratory tract (bronchi, bronchioles and alveoli) may have a strong negative impact on viral excretion. This may be due to physical obstruction of the deeper airways due to damage caused by H5N1 virus infection and immune responses. For pH1N1, it is more difficult to determine the main source of excreted virus, because virus antigen expression was high at all levels of the respiratory tract. However, the final GLM for pH1N1 viral excretion revealed that only the viral production in the nose and trachea was a significant predictor of viral excretion, suggesting that as for the H3N2 virus, the upper regions of the respiratory tract may be the main sources of excreted virus.

The pattern of H3N2 and H5N1 antigen expression in the ferret respiratory tract corresponded only in part with the pattern of attachment for these viruses (for the pH1N1 virus, the pattern of attachment in ferrets has not been determined). Attachment of an influenza virus to a host cell is the first step in the virus replication cycle and is considered to be necessary, but not sufficient, for subsequent infection of that cell. Previously, we found that H3N2 attached to many tracheal epithelial cells (predominantly ciliated cells), rare or few bronchial and bronchiolar epithelial cells, and a moderate number of alveolar epithelial cells (predominantly type I pneumocytes, which have a low metabolism) in the lower respiratory tract of the ferret [6]. However, in the current study, we found very little H3N2 infection (based on virus antigen expression) in any cell types of the ferret lower respiratory tract (Figure 3). This suggests that other factors were necessary for H3N2 to infect these cell types. These factors may be the virus load that may need to be higher for infection with H3N2 and the influence of surfactant that protect against influenza in humans and pigs [17]. We previously found that H5N1 did not attach to tracheal or bronchial epithelial cells, and attached to rare or few bronchiolar epithelial cells and a moderate number of alveolar epithelial cells (predominantly type II pneumocytes, which have a high metabolism) in the lower respiratory tract of the ferret [6]. In the current study, we found abundant H5N1 infection in alveolar and bronchiolar epithelial cells, which fits with the pattern of virus attachment (Figure 3). However, we also found H5N1 infection in tracheal and bronchial epithelial cells, which does not. Next to the fact that this could be related to a different H5N1 strain (A/Vietnam/1194/04 [6], while in the present experiment we used A/Indonesia/5/05), the replication of H5N1 in alveolar and bronchiolar epithelial cells was so abundant that tracheal and bronchial epithelial cells became infected despite the relatively weak attachment of H5N1 to the latter cell types.

Different parameters were used to measure the severity of lower respiratory tract disease in our ferrets. Of these parameters, relative lung weight and affected lung tissue allowed the best quantitative distinction between the virus groups: they showed clear differences between virus groups from 2 dpi onwards (Table 7), with relatively little within-group variation (Figure 2). These parameters have been used with success before for studies on severe acute respiratory syndrome [18] and on influenza [19]. Immunohistochemistry score for virus antigen expression in the alveoli (Figure 3) (Table 7), alveolar edema score, and alveolar hemorrhage score (Table 2) also showed clear differences between the virus groups. However, between-group differences differed according to the dpi, and within-group variation was large. Comparing the histopathology scoring for extent and severity of alveolitis and alveolar damage with the digital scoring for air-containing space in pulmonary tissue showed that the latter demonstrated less statistically significant results, and therefore is less useful for discriminating between viruses (Table 7). Comparing the antigen expression scoring in the alveoli by hand with the digital scoring and the virus titers, the *P*-values are similar and allow distinction between the virus groups (Table 7). The other parameters did not allow a clear distinction between the three virus groups.

**Table 7:** Statistical analyses comparing pathology and virology scores among ferrets inoculated with different influenza viruses.

		Significance of the difference (P-value)							
		Pathology scores				Virology scores			
Comparison	Day	Percentage of affected lung tissue	Relative lung weight (%)	Extent and severity of alveolitis and alveolar damage (Score 0–3)	Percentage of air containing space in pulmonary tissue (Digital scoring)	Virus titers lung tissue (log_10_ TCID_50_ per gram)	Antigen expression in the alveoli by immunohistochemistry (%)	Percentage of antigen expression in the alveoli (Digital scoring)	Number of antigen expression counts in the alveoli (Digital scoring)
H3N2 versus pH1N1	0.5	0.739	0.248	0.245	1.000	0.020*	0.019*	0.021*	0.020*
	1	0.017*	0.021*	0.019*	0.773	0.021*	0.018*	0.021*	0.018*
	2	0.018*	0.021*	0.019*	0.773	0.021*	0.018*	0.018*	0.017*
	3	0.013*	0.021*	0.021*	0.386	0.021*	0.017*	0.021*	0.076
	4	0.011*	0.021*	0.019*	0.021*	0.020*	0.014*	0.018*	0.026*
	7	0.014*	0.021*	0.019*	0.386	0.245	0.014*	0.772	0.439
	14	0.017*	0.021*	0.020*	0.564	0.309	1.000	0.508	0.508
H3N2 versus H5N1	0.5	0.036*	0.083	0.020*	0.386	0.020*	0.019*	0.021*	0.020*
	1	0.028*	0.083	0.019*	0.021*	0.021*	0.018*	0.021*	0.018*
	2	0.009**	0.011*	0.010*	0.019*	0.011*	0.009**	0.010*	0.010*
	3	0.019*	0.034*	0.032*	0.034*	0.034*	0.028*	0.034*	0.032*
	4	0.019*	0.034*	0.031*	0.077	0.032*	0.019*	0.028*	0.028*
pH1N1 versus H5N1	0.5	0.052	0.083	0.110	0.564	0.021*	0.083	0.386	0.386
	1	0.757	1.000	1.000	0.083	0.021*	0.083	0.043*	0.773
	2	0.009**	0.019*	0.010*	0.011*	0.831	0.054	0.201	0.032*
	3	0.048*	0.034*	0.285	0.034*	0.724	0.032*	0.034*	0.034*
	4	0.067	0.034*	0.064	0.034*	0.157	0.593	0.724	0.289

*P*-values were calculated by using the Mann-Whitney test.

* *P*-value<0.05.

** *P*-value<0.01.

Besides quantitative differences in severity of lower respiratory tract disease, the three virus infections also showed clear differences in the qualitative character of lower respiratory tract disease, related to the tropism of the virus and the ability of the host to counter infection. This is particularly well illustrated in the alveoli. For the mildest disease, caused by H3N2 infection, there was rare evidence of virus infection in the alveolar epithelium (Figure 3). There was a mild acute inflammatory response with small numbers of neutrophils and macrophages, peaking at 2 dpi. Damage to the alveolar epithelium was so mild that there was no visible evidence of epithelial cell hypertrophy or hyperplasia to re-epithelialize the alveolar walls (Table 2).

For the intermediate disease, caused by pH1N1 infection, virus infection already was high at 0.5 dpi and peaked soon afterwards at 1 dpi (Figure 3). There was a moderate acute inflammatory response characterized by influx of neutrophils, peaking at 1 dpi (Figure 9). At the same time, there was evidence of damage to the alveolar wall, characterized by necrosis of the epithelium and edema and hemorrhage in the alveolar lumina, which was maximal at 4 to 7 dpi (Table 2). After 1 dpi, the alveolar lesion progressed to a more chronic inflammatory response, with neutrophils being replaced by mononuclear cells, which peaked at 7 dpi (Figure 9). At the same time, repair of the alveolar epithelial damage was visible as hypertrophy and hyperplasia of type II pneumocytes, which was maximal at 7 dpi. During this period, the level of virus infection decreased to a low level. By 14 dpi the damage to and inflammation of the alveoli was completely resolved, except for a remnant of mononuclear cells in the alveolar lumina, and virus infection was no longer detectable. The resolution of edema and hemorrhage could be explained on the one hand by the action of alveolar macrophages [20], and on the other hand by re-epithelialization of the alveolar walls [21].

For the most severe disease, caused by H5N1 infection, the virus infection reached about the same level as that by pH1N1 infection at 1 dpi but remained high until death or euthanasia at 4 dpi, indicating that the host was not able to control virus infection. There was concurrent alveolar damage, which was so severe and extensive that all ferrets either died or had to be euthanized on humane grounds by 4 dpi. This was despite clear attempts at re-epithelialization, based on prominent type II pneumocyte hypertrophy and hyperplasia from 1 dpi onwards (Table 2). There was a marked acute inflammatory response characterized by neutrophil influx peaking at 1 dpi but much greater than in pH1N1 infection, with a neutrophil count that was nearly twice as high (Figure 9). This could be explained by a higher induction of pro-inflammatory cytokine production (e.g., IL-6 and IL-8) and NO production due to H5N1 infection [22], [23]. Maines et al. [10] demonstrated a relatively high production of IL-6 and IL-8 in the lower respiratory tract of ferrets infected with H5N1, but not with pH1N1. Similarly, humans infected with H5N1 had high levels of IL-6 and IL-8 in the blood [24]. Another major difference with pH1N1 infection was the low influx of mononuclear cells, with a mononuclear cell count of less than half that in pH1N1 infection by 4 dpi, suggesting that the inflammatory response never progressed beyond the acute stage.

Comparison of the dynamics of neutrophil and mononuclear cell influx in the alveolar lumina with neutrophil and monocyte counts in peripheral blood helps to explain the differences in hematological dynamics among the three virus infections (Figure 9). For H3N2 infection, the slight and transient increase in the numbers of neutrophils and monocytes in the blood at 1 dpi correlates with the mild influx of these cell types into the alveolar lumina at that time. This corresponds with a low demand for neutrophils and monocytes during H3N2 infection. For pH1N1 infection, the marked increase in first the number of neutrophils and then of monocytes in the blood correlates with the moderate influx of first neutrophils and then mononuclear cells in the alveolar lumina. This corresponds with increased demand for both cell types, both of which can be met adequately by the myelopoietic compartment. Neutrophils and monocytes are back to normal levels in the blood by 14 dpi, indicating resolution of the respiratory tract inflammation. For H5N1 infection, the initial increase of neutrophils in the blood at 1 dpi correlates with a massive influx of neutrophils into the alveolar lumina. The steep decline in neutrophils in the blood on subsequent days, despite continued high influx in alveolar lumina, corresponds to a demand that has outstripped the available supply by the myelopoietic compartment. This is corroborated by the presence of immature neutrophils with rod-shaped nuclei in the blood from 1 dpi onwards (Table 5). The lack of increase in monocytes in the blood corresponds with the failure of the acute inflammatory response in the lungs to transform into a more chronic inflammatory response. In addition to changes in neutrophil and monocyte counts, there was an overall leucopenia from 2 dpi (Table 5). Such a leucopenia has been described previously for H5N1 infection in both humans and ferrets [11], [12], [25], [26], whereas ferrets infected with pH1N1 showed higher leucocyte counts [12]. Together, this comparison between inflammatory cell dynamics in alveoli and blood provides insight into the interpretation of hematological analyses of influenza pneumonia.

Besides the respiratory tract, there was evidence, by virus isolation, of extra-respiratory spread for H5N1 and pH1N1, but not for H3N2 (Table 4). The failure to detect virus antigen in any of these virus-isolation-positive tissues except jejunum may be because virus isolation is more sensitive than immunohistochemistry, or because virus was present but not replicating in these tissues. Previously, H5N1 virus antigen expression in extra-respiratory tissue has been found in the central nervous system and liver of experimentally inoculated ferrets [27], [28], [29]. In our experiment, we additionally found virus antigen in other extra-respiratory tissues: for pH1N1 in sero-mucous glandular epithelial cells in the palatine roof, and for H5N1 in mononuclear cells in tracheo-bronchial lymph nodes, tonsils, sternal lymph node, spleen, and GALT of the jejunum, and in squamous stratified epithelium on the tip of the nose. The implication of these sites of replication for the pathogenesis and transmission of these viruses is yet to be elucidated.

The extensive H5N1 infection in extra-respiratory tissues known from previous studies [11], [28], [27], [30] likely occurs by spread from the respiratory tract via the blood [24], [31], [32]. However, the expression of H5N1 antigen in olfactory mucosa (Figure 5) together with high virus titres in the olfactory bulb (Table 4) suggest that H5N1 may reach the central nervous system from the nasal cavity via the olfactory route, as demonstrated recently by Schrauwen et al. [33]. This H5N1 infection of the central nervous system resulted in clinical disease, judging by the presentation of neurological signs (Table 1). In addition to extra-respiratory spread of H5N1, there also was evidence of pH1N1 in extra-respiratory tissues, especially the central nervous system (Table 4), and pH1N1 expression in olfactory mucosa (Figure 5). This suggests that pH1N1 also may be neurotropic and enter the central nervous system via the olfactory route.

## Methods

### Ethics statement

Animals were housed and experiments were conducted in strict compliance with European guidelines (EU directive on animal testing 86/609/EEC) and Dutch legislation (Experiments on Animals Act, 1997). The protocol was approved by the independent animal experimentation ethical review committee of the Netherlands Vaccine Institute (permit number 200900201) and was performed under animal biosafety level 3 conditions. Animal welfare was observed on a daily basis, and all animal handling was performed under light anesthesia using a mixture of ketamine and medetomidine to minimize animal suffering. After handling atipamezole was administered to antagonize the effect of medetomidine.

### Virus preparation

Three viruses were used: seasonal H3N2 virus (A/Netherlands/177/2008), isolated from a patient during the 2008 influenza season [34]; pandemic (H1N1) 2009 virus (pH1N1) (A/Netherlands/602/2009), isolated from a specimen of a human patient who had recently visited Mexico during the pandemic in 2009 [35]; and HPAI H5N1 virus (A/Indonesia/5/2005) as described earlier [35]. The H3N2 virus was chosen as a representative from a recent seasonal H3N2 influenza epidemic. Like virtually all recent H3N2 viruses the present virus exhibits the oseltamivir-sensitive neuraminidase-associated agglutination of turkey erythrocytes [36]. The pH1N1 virus which had been initially isolated in embryonated chicken eggs was chosen as a representative virus from the 2009 pandemic because it has been used in previous experiments by others [37] and ourselves [19], [35], [38]. The H5N1 virus was chosen as a representative of Clade 2 (subclade 1.3.2) of HPAI H5N1 virus, and has been used in several previous experiments [19], [29]. The different isolates were passaged three times in Madin-Darby Canine Kidney (MDCK) cells and titrated according to standard methods. Subsequently, the viruses were clarified and reached an infectious virus titer of 1×10^7.4^ median tissue culture infectious dose (TCID_50_) per ml for H3N2 virus, and 1×10^7.8^ TCID_50_ for both pH1N1 and H5N1 virus [8], [39].The inoculum for the control group was prepared as follows: MDCK cells were grown up to a monolayer of 80 to 90% in two 75 cm^2^ flasks (A and B). Virus medium (EMEM supplemented with HEPES, Sodium bicarbonate, BSA fraction V, L-glutamin, penicillin, streptomycin, trypsin and amphothericin-B) was added and the cells were incubated for 2 days at 37°C. To mimic cellular damage in MDCK cells during virus infection when preparing the virus stock we aimed to reach a cytopathic effect (CPE) of 75%. Therefore, the cells in bottle B were collected by disrupting the monolayer with 3 mm glass beads (VWR, Amsterdam, the Netherlands), sonication (3 times 20 seconds in melting ice) and freezing at −80°C, leading to lysis of all cells. The cells in bottle A were not damaged and the final inoculum consisted for 25% of the supernatant of bottle A and for 75% of the supernatant of bottle B.

### Study design

For every virus and the sham inoculated group, 7 groups (5 groups for H5N1) of four ferrets were inoculated under anesthesia with ketamine (4–8 mg/kg; Nimatek, Eurovet Animal Health B.V., Bladel, The Netherlands) and medetomidine hydrochloride (0.1 mg/kg; Domitor, Orion Pharma, Espoo, Finland) with each of these three viruses with 10^6^ TCID_50_ in a 3-ml volume intra-tracheally and in a 0.3-ml volume intranasally evenly divided over the two nostrils. Intratracheal and intranasal inoculation was performed to ensure that the inocula would reach both the lower and the upper respiratory tract [19], [29]. After inoculation, the ferrets received atipamezole hydrochloride (0.5 mg/kg Antisedan; Orion Pharma, Espoo Finland) and were monitored daily for clinical signs until maximally 14 dpi [8]. The animals were predestined to be sacrificed at 12 hours (0.5 day), 1, 2, 3, 4, 7 and 14 dpi (for H5N1 not at 7 and 14 dpi) in order to avoid any bias that could follow from clinical observation, or earlier when they were moribund before the selected time point of euthanasia (H5N1 only). The animals were euthanized by exsanguination after anesthesia with ketamine. At euthanasia, body weight was measured, nose, throat and rectal swabs were collected, blood was taken, and samples were taken from both respiratory and extra-respiratory tissues for virological, pathological, and immunohistochemical analyses.

The inoculum used for the sham control group induced comparable body weight loss, affected lung tissue, histological scores, digital scoring, and leucocyte counts as the H3N2 group. As an extra comparison, we therefore used non-inoculated healthy ferrets with the same age and background as the other ferrets as negative control animals to score and compare the above parameters.

### Ferrets

Ferrets were used in this experiment since they resemble disease in humans when infected with influenza A viruses [5], [7]. Hundred-and-four eleven-month-old purpose-bred ferrets, seronegative for antibodies against circulating influenza viruses H1N1 (A/Brisbane/059/2007), H3N2 (A/Uruguay/716/2007), and B: B/Brisbane/60/2008), and pH1N1 (A/Netherlands/602/2009), H5N1 (A/Indonesia/02/2005) and Aleutian disease virus, were maintained in standard housing, and provided with commercial food pellets and water. All ferrets were male (body weight: 1 302 to 2 150 g). Approximately three to four weeks prior to infection, the animals were anesthetized with ketamine and medetomidine hydrochloride, and a temperature logger (DST micro-T ultra small temperature logger; Star-Oddi, Reykjavik, Iceland) was placed in the peritoneal cavity. This device recorded the body temperature of the animals every 10 min. Effect of virus infection on body temperature was based on changes in the daily average of the maximum body temperatures of the ferrets per virus group.

Clinical scores in all groups were assessed every day. Activity status was scored as follows: 0, alert and playful; 1, alert and playful only when stimulated; 2, alert but not playful when stimulated; 3, neither alert nor playful when stimulated. For diarrhea, sneezing, nasal and conjunctival discharge, inappetence and dyspnea we scored: 0, not present; 1, present [11]. Inappetence was measured by the amount of food that was still present in the cages at the time of feeding. As a control we also assessed the amount of food that was present in the stomach and intestine of the animals on the day of necropsy. Dyspnea was characterized by open-mouth breathing with exaggerated abdominal movement. Additionally, we used four eleven-month-old purpose-bred ferrets, seronegative for antibodies against circulating influenza viruses H1N1 (A/Brisbane/059/2007), H3N2 (A/Uruguay/716/2007), and B: B/Brisbane/60/2008), and pH1N1 (A/Netherlands/602/2009), H5N1 (A/Indonesia/02/2005) and Aleutian disease virus. The four ferrets were euthanized immediately by the same method as described above and necropsied to provide control data of non-inoculated ferrets, and blood was taken blood for hematologic analyses and respiratory tract tissues for histopathological scoring.

### Pathology

For every virus four animals per time point were euthanized by exsanguination under ketamine/medetomidine anesthesia at 12 hours (0.5 day), 1, 2, 3, 4, 7 or 14 dpi and were necropsied according to a standard protocol. The trachea was clamped off to prevent the lungs from deflating upon opening the thoracic cavity, allowing visual estimation of the area of affected lung parenchyma. The lungs were weighed and the relative lung weight was calculated by the following formula: (lung weight on day of death/body weight on day of death)*100, and presented as percentage. The following tissues were collected for histological examination: left lung, left nasal concha, nasal septum, larynx, trachea, bronchus, tracheo-bronchial lymph node, left tonsil, heart, liver, spleen, kidney, pancreas, duodenum, jejunum, colon, adrenal gland, left olfactory bulb and left brain (cerebrum and cerebellum). Lung samples were taken in a standardized way, not guided by changes as seen in the gross pathology. Tissues were stored in 10% neutral-buffered formalin (lungs after careful inflation with formalin), embedded in paraffin, sectioned at 4 µm, and stained with hematoxylin and eosin (HE) for examination by light microscopy.

Semiquantitative assessment of influenza virus-associated inflammation in the lung (four slides with longitudinal section or cross-section of cranial or caudal lobes per animal) was performed on every slide as reported earlier [9]: for the extent of alveolitis and alveolar damage we used: 0, 0%; 1, 1–25%; 2, 25–50%; 3, >50%. For the severity of alveolitis, bronchiolitis, bronchitis, and tracheitis we scored: 0, no inflammatory cells; 1, few inflammatory cells; 2, moderate numbers of inflammatory cells; 3, many inflammatory cells. For the presence of alveolar edema, alveolar hemorrhage, and type II pneumocyte hyperplasia we scored: 0, no; 1, yes. Finally, for the extent of peribronchial, peribronchiolar, and perivascular infiltrates we scored: 0, none; 1, one to two cells thick; 2, three to ten cells thick; 3, more than ten cells thick. Slides were examined without knowledge of the treatment allocation of the animals. The cumulative scores for size and severity of inflammation of all slides provided the total score per animal.

To assess the number of neutrophils and alveolar macrophages in the alveolar lumina and the number of neutrophils in the alveolar walls, we counted them in 5 arbitrarily chosen 100× objective fields per HE-stained slide, with a total of 20 fields per animal. Neutrophils were identified on the basis of their size (approximately 12 to 15 µm in diameter) and the morphology of their nucleus (heterochromatic and segmented, with 3 to 5 lobes joined by thin strands). Neutrophils in the alveolar walls were counted when present in small capillaries, but were excluded if present in larger blood vessels. Pulmonary alveolar macrophages were identified on the basis of their morphology and location: large, oval to round cells with a distinct cell border, foamy cytoplasm and large, oval to bean-shaped nucleus, located in the alveolar lumen separate from the alveolar wall [40].

### Immunohistochemistry

For detection of influenza A virus antigen, tissues were stained with a primary antibody against the influenza A nucleoprotein as described previously [34]. In each staining procedure, an isotype control was included as a negative control and a lung section from a cat infected experimentally with H5N1 was used as positive control [41].

Semiquantitative assessment of influenza virus antigen expression in the lungs was performed as reported earlier [19]: for the alveoli, twenty-five arbitrarily chosen, 20× objective, fields of lung parenchyma of four lung sections were examined by light microscopy for the presence of influenza virus nucleoprotein, without the knowledge of the identity of the animals. The cumulative scores for each animal were presented as number of positive fields per 100 fields. For the nose, trachea, bronchi and bronchioles, the percentage of positively staining epithelium was estimated on every slide and the average of the four slides was taken to provide the score per animal: 0, 0%; 1, 1–25%; 2, 25–50%; 3, >50%.

For computerized scoring of slides we used the cross-section of the left caudal lung of all animals to make a digital scan using the NanoZoomer with accompanying software (NanoZoomer Digital Pathology and NDP.scan and NDP.view, Hamamatsu, Higashi-ku, Hamamatsu City, Japan). Of every scan, 20 pictures of the alveoli were made in a randomized order with the 20× objective. From every picture, we calculated: the quantity of tissue (visualized in blue staining) and the quantity of red staining consistent with virus antigen expression using Zeiss KS 400 version 3.0 image analysing system (Carl Zeiss Vision GmbH, Eching, Germany). The images were 1024×768 pixels with 0.455 µm per pixel. The percentage of air-containing space present in the pulmonary tissue was calculated as 100% minus the total percentage of pulmonary tissue (blue staining) that was present in the pictures. The quantity of virus replication was calculated either as the percentage of antigen expression (red staining) relative to quantity of tissue (blue staining), or as the number of times red staining was detected in a picture.

### Virology

After collection on day 0 and the day of euthanasia, nose, pharyngeal and rectal swabs were collected in virus transport medium (EMEM containing bovine serum albumin (fraction V), penicillin, streptomycin, amphothericin-B, L-glutamine, sodium bicarbonate and Hepes), aliquotted and stored at −70°C. Upon necropsy, samples were collected from the following tissues: nasal concha, trachea, bronchus, tracheo-bronchial lymph node, tonsil, heart, liver, spleen, kidney, pancreas, duodenum, jejunum, colon, adrenal gland, olfactory bulb and brain. Specifically from the lungs, sections of the cranial, median and caudal lobe of the right lung and of the accessory lobe from each animal were collected and pooled (total average weight of about 0.4–0.5 g/animal); lung samples were taken in a standardized way, not guided by changes as seen in the gross pathology. Tissue samples were homogenized with a FastPrep-24 (MP Biomedicals, Eindhoven, The Netherlands) in influenza infection medium (EMEM containing bovine serum albumin (fraction V), penicillin, streptomycin, amphothericin-B, L-glutamine, sodiumbicarbonate, Hepes and trypsin) and centrifuged briefly before titration. Quadruplicate 10-fold serial dilutions of tissue and swab supernatants were used to determine the virus titers in confluent layers of MDCK cells as described previously [39].

### Hematologic analyses

On day 0 and the day of euthanasia (0.5, 1, 2, 3, 4, 7 or 14 dpi or when an animal was sacrificed due to a moribund state) blood was collected from the euthanized animals. Total leucocyte counts were determined in blood collected in EDTA Vacutainer tubes (Vacuette, Greiner Bio-One GmbH, Kremsmünster, Austria) using an automated hematology analyser Sysmex pocH-100*i* (Sysmex Europe GMBH, Norderstedt, Germany). Thin blood films were prepared from EDTA blood and stained with May-Grünwald-Giemsa (Merck, Darmstadt, Germany). Differential cell counts were obtained by counting 100 cells per slide, and the numbers of lymphocytes, mononuclear cells, blastocytes, rod-shaped neutrophils, segmented neutrophils, eosinophils, basophils and normoblasts were calculated by multiplying these percentages by the leucocyte counts obtained for the same sample. By using the total leucocyte counts we calculated the absolute number of different leucocytes in 10^9^/L.

### Statistical analysis

The non-parametric Mann-Whitney test was used to compare several parameters of the different virus infection per day for; percentage of affected lung tissue, relative lung weight, extent and severity of alveolitis and alveolar damage, percentage of air containing space in pulmonary tissue, virus titers in lung tissue, antigen expression in the alveoli by immunohistochemistry, percentage of antigen expression in the alveoli by digital scoring, and number of antigen expression counts in the alveoli by digital scoring. The outcomes were considered significant when *P*<0.05.

Generalized linear models (GLM) were used to determine the most appropriate predictors of viral excretion for each virus, based on viral production and damage in the respiratory tract. These models were used to determine the linear equations of the form y = ∑(a_i_ x_i_+b_i_) that best fit the data, where y is viral excretion, x_i_ is a predictor, and a_i_ and b_i_ are the predictor and intercept coefficients, respectively. Viral excretion was measured as the sum of nasal swab and pharyngeal swab viral titers. Predictors initially included in the models were measures of viral production and measures of damage in upper (nose and trachea) and deeper regions of the respiratory tract (bronchi, bronchioles and alveoli), as well as all two-ways interactions. Measures of viral production for each region were calculated as the product of the viral titers and immunohistochemistry scores in the respective regions. This was done to take into account the fact that virus isolated in a particular region of the respiratory tract may not be produced locally. Measures of damage for each region were calculated as the sum of the severity scores in the respective regions. The severity scores were used because they were similarly assessed in all regions of the respiratory tract. Only predictors or interactions of predictors with *P*<0.05 were retained in the final GLM.

## Supporting Information

Supporting Information S1 Detailed description per virus of histopathologic changes and antigen expression by immunohistochemistry.
(DOC)

## References

[1] Kuiken T, Taubenberger JK (2008) Pathology of human influenza revisited. Vaccine 26 Suppl 4: D59–D66. 19230162 10.1016/j.vaccine.2008.07.025 PMC2605683

[2] Guarner J, Falcon-Escobedo R (2009) Comparison of the pathology caused by H1N1, H5N1, and H3N2 influenza viruses. Arch Med Res 40: 655–661. 20304252 10.1016/j.arcmed.2009.10.001

[3] Taubenberger JK, Morens DM (2008) The pathology of influenza virus infections. Annu Rev Pathol 3: 499–522. 18039138 10.1146/annurev.pathmechdis.3.121806.154316 PMC2504709

[4] WHO (2012) Number of human H5N1 cases. Available: http://www.who.int/influenza/human_animal_interface/EN_GIP_20120405CumulativeNumberH5N1cases.pdf. Accessed 6 April 2012.

[5] Kuiken T, van den Brand JM, van Riel D, Pantin-Jackwood M, Swayne DE (2010) Comparative pathology of select agent influenza a virus infections. Vet Pathol 47: 893–914. 20682805 10.1177/0300985810378651

[6] van Riel D, Munster VJ, de Wit E, Rimmelzwaan GF, Fouchier RA, et al (2007) Human and avian influenza viruses target different cells in the lower respiratory tract of humans and other mammals. Am J Pathol 171: 1215–1223. 17717141 10.2353/ajpath.2007.070248 PMC1988871

[7] Maher JA, DeStefano J (2004) The ferret: an animal model to study influenza virus. Lab Anim (NY) 33: 50–53. 15457202 10.1038/laban1004-50

[8] Baras B, Stittelaar KJ, Simon JH, Thoolen RJ, Mossman SP, et al (2008) Cross-protection against lethal H5N1 challenge in ferrets with an adjuvanted pandemic influenza vaccine. PLoS ONE 3: e1401 –doi:10.1371/journal.pone.0001401. 18167560 10.1371/journal.pone.0001401 PMC2151135

[9] van den Brand JM, Kreijtz JH, Bodewes R, Stittelaar KJ, van Amerongen G, et al (2011) Efficacy of vaccination with different combinations of MF59-adjuvanted and non-adjuvanted seasonal and pandemic influenza vaccines against pandemic H1N1 (2009) influenza in ferrets. J Virol 85: 2851–2858. 21209108 10.1128/JVI.01939-10 PMC3067945

[10] Maines TR, Belser JA, Gustin KM, Van Hoeven N, Zeng H, et al (2012) Local innate immune responses and influenza virus transmission and virulence in ferrets. J Infect Dis 205: 474–485. 22158704 10.1093/infdis/jir768

[11] Zitzow LA, Rowe T, Morken T, Shieh WJ, Zaki S, et al (2002) Pathogenesis of avian influenza A (H5N1) viruses in ferrets. J Virol 76: 4420–4429. 11932409 10.1128/JVI.76.9.4420-4429.2002 PMC155091

[12] Belser JA, Gustin KM, Maines TR, Blau DM, Zaki SR, et al (2011) Pathogenesis and transmission of triple-reassortant swine H1N1 influenza viruses isolated before the 2009 H1N1 pandemic. J Virol 85: 1563–1572. 21123386 10.1128/JVI.02231-10 PMC3028905

[13] Rowe T, Leon AJ, Crevar CJ, Carter DM, Xu L, et al (2010) Modeling host responses in ferrets during A/California/07/2009 influenza infection. Virol 401: 257–265. 10.1016/j.virol.2010.02.020 PMC2862141 20334888

[14] McBrayer A, Camp JV, Tapp R, Yamshchikov V, Grimes S, et al (2010) Course of seasonal influenza A/Brisbane/59/07 H1N1 infection in the ferret. Virol J 7: 149 –doi: 10.1186/1743-422X-7-149. 20618974 10.1186/1743-422X-7-149 PMC2909963

[15] Smith JH, Nagy T, Driskell E, Brooks P, Tompkins SM, et al (2011) Comparative pathology in ferrets infected with H1N1 influenza A viruses isolated from different hosts. J Virol 85: 7572–7581. 21593156 10.1128/JVI.00512-11 PMC3147907

[16] Meunier I, Embury-Hyatt C, Stebner S, Gray M, Bastien N, et al (2012) Virulence differences of closely related pandemic 2009 H1N1 isolates correlate with increased inflammatory responses in ferrets. Virol 422: 125–131. 10.1016/j.virol.2011.10.018 22074911

[17] Benne CA, Kraaijeveld CA, van Strijp JA, Brouwer E, Harmsen M, et al (1995) Interactions of surfactant protein A with influenza A viruses: binding and neutralization. J Infect Dis 171: 335–341. 7844369 10.1093/infdis/171.2.335

[18] Haagmans BL, Kuiken T, Martina BE, Fouchier RA, Rimmelzwaan GF, et al (2004) Pegylated interferon-alpha protects type 1 pneumocytes against SARS coronavirus infection in macaques. Nat Med 10: 290–293. 14981511 10.1038/nm1001 PMC7095986

[19] van den Brand JM, Stittelaar KJ, van Amerongen G, Rimmelzwaan GF, Simon J, et al (2010) Severity of pneumonia due to new H1N1 influenza virus in ferrets is intermediate between that due to seasonal H1N1 virus and highly pathogenic avian influenza H5N1 virus. J Infect Dis 201: 993–999. 20187747 10.1086/651132 PMC7110095

[20] Berthiaume Y, Matthay MA (2007) Alveolar edema fluid clearance and acute lung injury. Respir Physiol Neurobiol 159: 350–359. 17604701 10.1016/j.resp.2007.05.010 PMC2682357

[21] Folkesson HG, Nitenberg G, Oliver BL, Jayr C, Albertine KH, et al (1998) Upregulation of alveolar epithelial fluid transport after subacute lung injury in rats from bleomycin. Am J Physiol 275: L478–L490. 9728042 10.1152/ajplung.1998.275.3.L478

[22] Karpuzoglu E, Ahmed SA (2006) Estrogen regulation of nitric oxide and inducible nitric oxide synthase (iNOS) in immune cells: implications for immunity, autoimmune diseases, and apoptosis. Nitric Oxide 15: 177–186. 16647869 10.1016/j.niox.2006.03.009

[23] Teijaro JR, Walsh KB, Cahalan S, Fremgen DM, Roberts E, et al (2011) Endothelial cells are central orchestrators of cytokine amplification during influenza virus infection. Cell 146: 980–991. 21925319 10.1016/j.cell.2011.08.015 PMC3176439

[24] de Jong MD, Simmons CP, Tran TT, Hien VM, Smith GJ, et al (2006) Fatal outcome of human influenza A (H5N1) is associated with high viral load and hypercytokinemia. Nat Med 12: 1203–1207. 16964257 10.1038/nm1477 PMC4333202

[25] Oner AF, Bay A, Arslan S, Akdeniz H, Sahin HA, et al (2006) Avian influenza A (H5N1) infection in eastern Turkey in 2006. N Engl J Med 355: 2179–2185. 17124015 10.1056/NEJMoa060601

[26] Abdel-Ghafar AN, Chotpitayasunondh T, Gao Z, Hayden FG, Nguyen DH, et al (2008) Update on avian influenza A (H5N1) virus infection in humans. N Engl J Med 358: 261–273. 18199865 10.1056/NEJMra0707279

[27] Maines TR, Lu XH, Erb SM, Edwards L, Guarner J, et al (2005) Avian influenza (H5N1) viruses isolated from humans in Asia in 2004 exhibit increased virulence in mammals. J Virol 79: 11788–11800. 16140756 10.1128/JVI.79.18.11788-11800.2005 PMC1212624

[28] Yen HL, Lipatov AS, Ilyushina NA, Govorkova EA, Franks J, et al (2007) Inefficient transmission of H5N1 influenza viruses in a ferret contact model. J Virol 81: 6890–6898. 17459930 10.1128/JVI.00170-07 PMC1933302

[29] Bodewes R, Kreijtz JH, van Amerongen G, Fouchier RA, Osterhaus AD, et al (2011) Pathogenesis of Influenza A/H5N1 virus infection in ferrets differs between intranasal and intratracheal routes of inoculation. Am J Pathol 179: 30–36. 21640972 10.1016/j.ajpath.2011.03.026 PMC3123863

[30] Govorkova EA, Rehg JE, Krauss S, Yen HL, Guan Y, et al (2005) Lethality to ferrets of H5N1 influenza viruses isolated from humans and poultry in 2004. J Virol 79: 2191–2198. 15681421 10.1128/JVI.79.4.2191-2198.2005 PMC546577

[31] Tumpey TM, Lu X, Morken T, Zaki SRKatz JM (2000) Depletion of lymphocytes and diminished cytokine production in mice infected with a highly virulent influenza A (H5N1) virus isolated from humans. J Virol 74: 6105–6116. 10846094 10.1128/jvi.74.13.6105-6116.2000 PMC112109

[32] Rimmelzwaan G, van Riel D, Baars M, Bestebroer TM, van Amerongen G, et al (2006) Influenza A virus (H5N1) infection in cats causes systemic disease with potential novel routes of virus spread within and between hosts. Am J Pathol 168: 176–183. 16400021 10.2353/ajpath.2006.050466 PMC1592682

[33] Schrauwen EJ, Herfst S, Leijten LM, van Run P, Bestebroer TM, et al (2012) The multibasic cleavage site in H5N1 virus is critical for systemic spread along the olfactory and hematogenous routes in ferrets. J Virol 86: 3975–3984. 22278228 10.1128/JVI.06828-11 PMC3302532

[34] van den Brand JM, Stittelaar KJ, Leijten LM, van Amerongen G, Simon JH, et al (2012) Modification of the ferret model for pneumonia from seasonal human influenza A virus infection. Vet Pathol 49: 562–568. 22262355 10.1177/0300985811429812

[35] Munster VJ, de Wit E, van den Brand JM, Herfst S, Schrauwen EJ, et al (2009) Pathogenesis and transmission of swine-origin 2009 A(H1N1) influenza virus in ferrets. Science 325: 481–483. 19574348 10.1126/science.1177127 PMC4814155

[36] Lin YP, Gregory V, Collins P, Kloess J, Wharton S, et al (2010) Neuraminidase receptor binding variants of human influenza A(H3N2) viruses resulting from substitution of aspartic acid 151 in the catalytic site: a role in virus attachment? J Virol 84: 6769–6781. 20410266 10.1128/JVI.00458-10 PMC2903250

[37] Itoh Y, Shinya K, Kiso M, Watanabe T, Sakoda Y, et al (2009) In vitro and in vivo characterization of new swine-origin H1N1 influenza viruses. Nature 460: 1021–1025. 19672242 10.1038/nature08260 PMC2748827

[38] Baras B, de Waal L, Stittelaar KJ, Jacob V, Giannini S, et al (2011) Pandemic H1N1 vaccine requires the use of an adjuvant to protect against challenge in naive ferrets. Vaccine 29: 2120–2126. 21238573 10.1016/j.vaccine.2010.12.125

[39] Rimmelzwaan GF, Baars M, Claas EC, Osterhaus AD (1998) Comparison of RNA hybridization, hemagglutination assay, titration of infectious virus and immunofluorescence as methods for monitoring influenza virus replication in vitro. Journal of Virological Methods 74: 57–66. 9763129 10.1016/s0166-0934(98)00071-8

[40] Dellmann HD (1993) Textbook of veterinary histology. Philadelphia: Lea & Febiger 56–149.

[41] Kuiken T, Rimmelzwaan G, van Riel D, van Amerongen G, Baars M, et al (2004) Avian H5N1 influenza in cats. Science 306: 241. 15345779 10.1126/science.1102287

