# Supplementary material for: Comparison of Temporal and Spatial Dynamics of Seasonal H3N2, Pandemic H1N1 and Highly Pathogenic Avian Influenza H5N1 Virus Infections in Ferrets
Source: PLoS One. 2012 Aug 8;7(8):e42343. doi: 10.1371/journal.pone.0042343 (PMC3414522; doi:10.1371/journal.pone.0042343)
Supplement: Supporting Information S1 — Detailed description per virus of histopathologic changes and antigen expression by immunohistochemistry. (DOC) [file pone.0042343.s001.doc]

**Supporting information S1.** Detailed description per virus of histopathologic changes and antigen expression by immunohistochemistry.

**Seasonal H3N2**

On 0.5 dpi, no lesions were seen in the nasal concha and immunohistochemistry showed few cells (scored as < 25 % of the total nasal epithelium) expressing viral antigen in the respiratory nasal epithelium (Figure 3). In the trachea, bronchi and bronchioles there were few intra-epithelial neutrophils and focal mild peribronchiolar and perivascular infiltration (cuffing) of few lymphocytes and plasma cells of 1 to 2 cells thick. No antigen expression was seen in the tracheal, bronchial, bronchiolar, and tracheo-bronchial glandular epithelium at any time point. The alveoli showed mild multifocal alveolitis, characterized by mild infiltration of few neutrophils in the alveolar walls (Table 2) and few neutrophils, mononuclear cells (believed to be alveolar macrophages) (Figure 9) and a small amount of edema in the alveolar lumina. The extent of the alveolitis was between 0 and 50 % of the lung tissue present in the slides (Table 2). Semiquantitative scoring showed antigen expression in 0 to 2 % of the type II pneumocytes in the alveoli examined.

On 1 dpi, similar lesions were seen. Additionally, there was a mild rhinitis with infiltration of few neutrophils in the respiratory epithelium accompanied by a slight increase in antigen expressing cells (in between 25-50 %) and also virus antigen expression in a few cells of the olfactory epithelium (Figure 4 and 5). In the alveoli, there was focal mild necrosis of the lining epithelium but no edema (Figure 8). One animal had a severe multifocal chronic granulomatous pleuropneumonia that was not related to the influenza virus infection.

On 2 dpi, there was a slight decrease in the number of antigen expressing cells in the nose. There was a mild broncho-adenitis with infiltration of few neutrophils, macrophages and mild necrosis of glandular epithelial cells and a mild increase of the numbers of neutrophils in the alveolar walls (Table 2).

On 3 dpi, there were few to moderate numbers of neutrophils in the nasal respiratory epithelium and extending into the lamina propria, with mild interstitial edema and mild necrosis as well as regeneration of the epithelium. In the bronchi, there was no broncho-adenitis. In the alveoli, a small amount of edema was seen and there were no neutrophils in the alveolar lumina.

On 4 dpi, the severity of the rhinitis increased with moderate numbers of neutrophils, and antigen was seen in < 25 % of the epithelium. In the alveoli, the number of neutrophils in the walls slightly increased as compared to 3 dpi (Table 2).

On 7 dpi, there were many neutrophils but no antigen expression in the nose (Figure 3, 4 and 5). In the lungs, there was a mild broncho-adenitis.

On 14 dpi, the extent of the lesions in the alveoli was < 25 % with no neutrophils in the alveolar lumen and few neutrophils in the alveolar walls.

**pH1N1**

On 0.5 dpi the lesions were limited to the following sites. The nasal concha showed a mild rhinitis with few neutrophils in the nasal epithelium. By immunohistochemistry, there was antigen expression in 25 to 50 % of the single layered nasal respiratory epithelial cells (Figures 3 and 4). In the trachea, there was a mild multifocal tracheitis with mild infiltration of neutrophils in the wall and lumen (and occasionally in the tracheal glands) that lasted until 14 dpi. The bronchial and bronchiolar epithelium had mild infiltration of few neutrophils. In peribronchiolar and to a lesser extent peribronchial and perivascular tissues, there was infiltration of few cells (1 to 2 cells thick), consisting predominantly of macrophages and lymphocytes, with few neutrophils and plasma cells. In the bronchi, there was antigen expression in < 25 % of the epithelium and in the bronchioles it was between 25 and 50 %. Bronchial glandular epithelial cells expressed virus antigen with often more expression in glandular cells than in bronchial epithelial cells. The alveoli showed diffuse alveolar damage (DAD) (Figure 8 and 10) that was characterized by an alveolitis with a moderate number of neutrophils in the alveolar walls (Table 2) and occasional neutrophils, few alveolar macrophages, and few eosinophils in the alveolar lumina (Figure 9). The extent of the alveolar lesions was between 25 and 50 % of the pulmonary tissue on the slides. Influenza virus antigen expression was closely associated with the presence of histological lesions and was seen predominantly in type II pneumocytes and less in type I pneumocytes and alveolar macrophages (Figure 8). Semi-quantitative scoring of the alveoli showed that the percentage of antigen expressing cells was high (Figure 3).

On 1 dpi, the numbers of neutrophils in the nose had increased and antigen expression was present in > 50 % of the respiratory epithelium and was also seen in the basal and olfactory cells of the olfactory epithelium (Figures 3 and 5). The inflammatory infiltrate in bronchial and bronchiolar epithelium was increased to many neutrophils, and necrosis of the epithelium that was more pronounced in the bronchioles when compared to the bronchi. There was cellular debris in the bronchiolar lumina. There were many inflammatory cells, predominantly macrophages and lymphocytes, with few neutrophils and plasma cells, in the bronchiolar epithelium. Peribronchial, peribronchiolar and perivascular cuffing was increased to 3 to 10 cells thick, and consisted predominantly of macrophages and lymphocytes, with few neutrophils and plasma cells. Broncho-adenitis was present. There was antigen expression in > 50 % of the bronchiolar epithelium and in 25 to 50 % of the bronchial epithelium. In the alveolar walls and lumina, there were more neutrophils, and in the alveolar lumina there were more mononuclear cells mixed with scant edema fluid, fibrin, erythrocytes, and cellular debris. The alveolar walls were thickened, had mild necrosis of the lining epithelium, and mild type II pneumocyte hypertrophy and hyperplasia. There was a mild to severe broncho-adenitis. Immunohistochemistry scores showed a peak in the number of antigen expressing cells in both upper and lower respiratory tract.

On 2 dpi in the bronchioles, necrosis was more severe, in some locations with complete absence of bronchiolar epithelial cells, clearly present denuded basement membranes and large amounts of cellular debris mixed with alveolar macrophages, neutrophils, erythrocytes, fibrin and edema fluid in the lumina. There was regeneration of bronchiolar epithelial cells with hyperplasia and hypertrophy, and moderate broncho-adenitis. Antigen expression in the bronchioles was decreased to between 25 and 50 %. In the alveoli, the severity of the lesions had increased, with more epithelial necrosis, edema, hemorrhage and type II pneumocyte hyperplasia. Semi-quantitative scoring of the alveoli showed that the percentage of antigen expressing cells had decreased (Figure 3), possibly due to necrosis of antigen-expressing epithelial cells.

On 3 dpi, there was regeneration of the nasal epithelium, characterized by hypertrophy and hyperplasia of epithelial cells. In the trachea, there was expression of antigen in epithelial cells. In the bronchi and bronchioles, there was more epithelial regeneration, and infiltration with many inflammatory cells. In the alveoli, it was remarkable that there were both new lesions with acute necrosis and old lesions with type II hyperplasia present; the amount of edema and hemorrhage as well as type II hyperplasia were increased. The number of neutrophils in the lumina was slightly decreased but the number of mononuclear cells was increased (Figure 9). By semi-quantitative scoring, the percentage of antigen-expressing cells in the alveoli had decreased again (Figure 3).

On 4 dpi, the number of antigen expressing bronchial epithelial cells had decreased to < 25 %. In the alveoli, both old and new lesions were present with increased amount of edema, hemorrhage, hyperplasia and increased numbers of mononuclear cells (Table 2 and Figure 8). Antigen expression was less than on 3 dpi for all tissues except the alveoli were there were more antigen expressing cells (Figure 3).

On 7 dpi, antigen expression in the nose had decreased and was absent in the tracheal epithelium. Peribronchial, peribronchiolar and perivascular cuffing was increased to sometimes more than 10 cells thick. Antigen expression scoring was lower in bronchi and bronchioles, and there was severe broncho-adenitis. In the alveoli, there was more edema, hemorrhage, necrosis and type II hyperplasia, often around bronchi and bronchioles. The number of mononuclear cells and neutrophils in the lumina showed the highest values (Figure 9). Antigen expression decreased further with only few positive cells (Figure 3).

On 14 dpi, there were few inflammatory cells in the nose. In the bronchi and bronchioles, there were few inflammatory cells and peribronchial, peribronchiolar and perivascular cuffing was between 3 to 10 cells thick. The numbers of neutrophils in the alveolar walls and lumina were comparable to those on 0.5 dpi, and the number of mononuclear cells in the lumina was decreased (Figure 9). The extent of the lesions was between 25 and 50 %. No antigen expression was seen in any of the respiratory tissues (Figure 3).

In the tracheo-bronchial lymph nodes, there was mild lymphoid hyperplasia on 0.5 dpi. Additionally, on 4 dpi, there was multifocal accumulation of proteinaceous fluid, histiocytosis and infiltration of mononuclear cells in the surrounding tissue, and on 14 dpi there was also severe lymphocytolysis. In the tonsils, there was mild lymphoid hyperplasia with few intra-epithelial lymphocytes and neutrophils with mild lymphocytolysis on 1 dpi. On 2 and 3 dpi, there were slightly more inflammatory cells, which decreased in number on 4 dpi. On 14 dpi, the tonsillar tissue was again normal. Tracheo-bronchial lymph nodes and tonsils did not express virus antigen. In the palatine roof of two animals on 7 dpi, there was a severe adenitis of the sero-mucous glands with necrosis of glandular epithelial cells, infiltration by many neutrophils, dilatation of glands, and antigen expression in the glandular epithelial cells. There was no virus antigen expression in other extra-respiratory tissues.

**H5N1**

On 0.5 dpi, the nose showed a moderate rhinitis with necrosis of epithelial cells and infiltration of a moderate number of neutrophils in the lumen, epithelium, and lamina propria. There was no virus antigen expression. In the trachea, there was a mild multifocal tracheitis with infiltration of few neutrophils in the wall and lumen, and tracheal glands. By immunohistochemistry, few tracheal epithelial cells presented virus antigen (Figure 3). The bronchial and bronchiolar epithelium had multifocal infiltration with moderate numbers of neutrophils and mild epithelial necrosis. There were peribronchial, peribronchiolar, and perivascular infiltrates (cuffing), 3 to 10 cells thick, consisting of few macrophages, lymphocytes, neutrophils and plasma cells. Bronchial and bronchiolar lumina contained small amounts of cellular debris, fibrin, and edema fluid, mixed with few neutrophils. Overall, changes in the bronchi were milder than in the bronchioles. There was a focally mild broncho-adenitis. By immunohistochemistry, virus antigen expression was present in < 25 % of the bronchiolar epithelial cells and not in the bronchial epithelial cells. The alveoli showed DAD (Figure 8) characterized by few neutrophils, eosinophils, and erythrocytes, and a moderate number of mononuclear cells, mixed with scant edema fluid in the alveolar lumina, and a moderate number of neutrophils in the alveolar walls (Table 2). The alveolar walls were mildly thickened and showed mild necrosis of epithelial cells and mild epithelial hypertrophy. The extent of the alveolar lesions was between 25 and 50 %. Semi-quantitative scoring showed that the extent and severity of the lesions was high (Table 2). Virus antigen expression was closely associated with the presence of histological lesions and was seen predominantly in type II pneumocytes (Figure 8), and less in type I pneumocytes and alveolar macrophages. Semiquantitative scoring for virus antigen expression in the alveoli was high (Figure 3).

On 1 dpi, the number of inflammatory cells in the nose was decreased. By immunohistochemistry, virus antigen expression occurred occasionally in the olfactory epithelium (with cytoplasmic staining towards the lumen), in respiratory epithelial cells and cells in the lamina propria (presumably glandular cells). The extent of the antigen expression was < 25 %. There were moderate to high numbers of neutrophils in the bronchiolar walls and lumina with increased epithelial necrosis. The number of neutrophils in the alveolar walls was increased with severe epithelial necrosis, and there were increased numbers of neutrophils and mononuclear cells with more edema fluid mixed with fibrin, necrotic neutrophils, cellular debris, and erythrocytes in the alveolar lumina. Semi-quantitative scoring for virus antigen expression was increased in the trachea and alveoli.

On 2 dpi, virus antigen expression in the nose was between 25 and 50 %. Bronchial epithelium showed < 25 % antigen expression. In the bronchioles, there was hypertrophy and hyperplasia of bronchiolar epithelium, as well as inflammation. In the alveoli, there was hypertrophy and hyperplasia of type II pneumocytes, and slightly fewer neutrophils and mononuclear cells (Table 2 and Figure 9). There were high scores for the presence of edema fluid, hemorrhage and type II hypertrophy and hyperplasia.

On 3 dpi the extent of the antigen expression was between 25 and 50 % in the nose and bronchioles and < 25 % in the bronchi. In the alveoli, there was severe necrosis, and slightly decreased numbers of neutrophils and mononuclear cells.

On 4 dpi the extent of the antigen expression in the nose was < 25 %. In the trachea, the scores for tracheitis were higher than on previous days. In the bronchioles, there was severe epithelial necrosis as well as epithelial hypertrophy and hyperplasia. There was broncho-adenitis with only rare antigen expression. In the alveoli, epithelial necrosis was very severe, the number of neutrophils in the walls was decreased, and the number of mononuclear cells in the lumina was increased. There was severe interstitial and pleural edema and hemorrhage and the extent of the lesions was > 50 %. Semiquantitative scoring for antigen expression in the alveoli was decreased. This severe drop in antigen expressing cells could be explained by the severe necrosis.

In the tracheo-bronchial lymph nodes, there was mild lymphoid hyperplasia on 0.5 dpi. On 1 dpi, there was moderate lymphoid hyperplasia and histiocytosis, multifocal accumulation of proteinaceous fluid, infiltration of neutrophils, lymphocytolysis and antigen expressing mononuclear cells in the perifollicular area. On 3 dpi additionally there was severe multifocal necrosis and associated antigen expression, and on 4 dpi there was lymphoid depletion with decreased numbers of follicles and no antigen expression. In the tonsils, there was mild lymphoid hyperplasia with few intra-epithelial neutrophils and few virus-antigen-expressing cells (macrophage-like) on 2 dpi. On 3 dpi, there was multifocal necrosis and many intra-epithelial neutrophils, and antigen expression in few mononuclear cells and on 4 dpi there was little necrosis and little antigen expression. In one animal, there was virus antigen expression in squamous stratified epithelium in the tip of the nose on 2 dpi, with associated intraepithelial neutrophils and erosion of the epithelium. In the extra-respiratory tissues, one animal had few virus antigen expressing mononuclear cells in the sternal lymph node on 2 dpi, associated with mild lymphoid hyperplasia, and another animal had virus antigen expression in endothelial cells of the soft palate on 1 dpi associated with severe necrotizing and neutrophilic pharyngitis. Two animals had virus antigen expression in the gut associated lymphoid tissue (GALT) of the jejunum on 2 and 3 dpi, and associated lymphoid hyperplasia, mild necrosis, multifocal accumulation of proteinaceous material and neutrophils. One animal had few virus antigen expressing mononuclear cells in the spleen on 4 dpi, and associated necrosis, protein deposits and mild lymphoid depletion.

**Control group with inoculum**

On 0.5 dpi, there was a mild rhinitis and tracheitis with few inflammatory cells. Almost all animals had a mild bronchitis or bronchiolitis with few neutrophils. There was perivascular and peribronchiolar cuffing (1-2 cells thick) with few neutrophils, lymphocytes, plasma cells and macrophages. In the alveoli, there was a mild focal alveolitis with infiltration of few neutrophils and eosinophils in the alveolar septa and in the alveolar lumina occasionally few neutrophils, mononuclear cells and eosinophils with small amounts of edema fluid and few erythrocytes. In few animals, there was a mild broncho-adenitis with few neutrophils, lymphocytes and plasma cells in the bronchial glands.

On 1 dpi, there was a slight increase in the number of eosinophils and slightly more edema fluid in the alveoli.

On 2 dpi, perivascular and peribronchiolar cuffing was up to 10 cells thick. One ferret had multifocal moderate neutrophilic infiltrates mixed with edema fluid in the alveoli and associated terminal bronchioles. This was considered an incidental finding.

On 7 dpi, the number of neutrophils in the alveolar walls was higher than on other days.

No virus antigen expression was seen in any of the tissues.
